# Molecular modeling design of antitrypanosomal pyrazolone derivatives targeting Chagas disease: QSAR, docking, molecular dynamics and free energy calculations

**DOI:** 10.1007/s00894-026-06923-0

**Published:** 2026-09-09

**Authors:** Karen Karla F. Sousa, Ana Beatriz S. Barbosa, Fábio José B. Cardoso, José Rogério A. Silva, Fábio A. Molfetta

**Affiliations:** 1 Laboratório de Modelagem Molecular, Instituto de Ciências Exatas e Naturais, Universidade Federal do Pará, Belém, 66075-110 Brazil; 2 Laboratory of Computer Modeling of Molecular Biosystems (CompMBio), Federal University of Pará, Belém, 66075-110 Brazil; 3 Catalysis and Peptide Research Unit, University of KwaZulu-Natal, Durban, 4000 South Africa

**Keywords:** Chagas disease, Cruzain, Pyrazolone derivatives, 2D-QSAR, Molecular dynamics, MM/GBSA, Drug-likeness

## Abstract

**Context:**

Chagas disease, caused by *T. cruzi*, remains a neglected tropical disease with limited therapeutic options, underscoring the urgent need for novel antitrypanosomal agents. A computer-aided drug design (CADD) protocol was applied to investigate pyrazolone derivatives with reported antitrypanosomal activity and their putative interaction with cruzain (Cz), a cysteine protease essential for parasite survival. A 2D-QSAR model built from a curated set of 33 phenyl-dihydropyrazolone derivatives guided the design of four new candidates (LMM1–LMM4), which showed intermediate predicted antitrypanosomal activity within the chemical space of the original pyrazolone series. Molecular docking identified favorable binding within the Cz active site, with contacts at residues Ser61, Gly66, and Leu67. MD simulations indicated overall structural stability of the protein–ligand complexes and revealed ligand-dependent conformational behavior within the Cz binding site. MM/GBSA calculations suggested that van der Waals interactions are major contributors to the computed binding energies; however, the MM/GBSA ranking did not fully reproduce the phenotypic antitrypanosomal activity trend. Per-residue decomposition and free energy landscape analyses were therefore interpreted qualitatively, suggesting that contacts with S2/S3 subsite residues and conformational adaptability may contribute to Cz recognition but do not, alone, explain whole-cell potency. These findings support pyrazolones as antitrypanosomal scaffolds and provide structural hypotheses for future experimental validation against Cz.

**Methods:**

Molecular geometries were optimized at the PM6 level (MOPAC2016); descriptors were calculated with ChemDes and UseGalaxy, selected via OPS and a genetic algorithm (QSARINS), and the QSAR model was built using PLS regression (QSAR modeling software). Molecular docking was performed with GOLD, FITTED, and AutoDock Vina 1.2.3 against Cz (PDB: 3KKU). ADMET properties were predicted using OSIRIS Property Explorer, SwissADME, and ADMET-AI. Partial atomic charges were derived at the HF/6-31G* level with RESP fitting (Gaussian 09); MD simulations (3 × 250 ns per system) were run with AMBER20 using GAFF/ff14SB force fields and TIP3P solvent. Binding free energies were calculated by MM/GBSA (MMPBSA.py).

**Supplementary information:**

The online version contains supplementary material available at https://doi.org/10.1007/s00894-026-06923-0.

## Introduction

Chagas disease (CD), caused by the protozoan parasite *Trypanosoma cruzi* (*T. cruzi*), remains a major neglected tropical disease in the Americas, disproportionately affecting vulnerable populations and placing more than 70 million people at risk of infection [1, 2]. The combined effects of human migration, urbanization, and climate change have driven the spread of CD beyond traditionally endemic areas, with increasing numbers of cases now reported in Europe, North America, Japan, and Australia [3, 4]. Transmission occurs through complex domestic, peridomestic, and sylvatic cycles involving multiple mammalian reservoirs and blood-feeding triatomine vectors (“kissing bugs”), which contaminate the skin or mucosal surfaces with infective trypomastigotes in their feces; additional routes include oral, congenital, transfusional, transplant-associated, and accidental laboratory exposure [5–9]. The absence of an effective vaccine, combined with continued reliance on the old nitroheterocyclic drugs nifurtimox and benznidazole, both associated with significant adverse effects and limited efficacy, particularly in the chronic phase of the disease, underscores the urgent need for new therapeutic alternatives [10].

Pyrazolones, heterocyclic compounds derived from the pyrazole core, are widely used as pharmaceutical scaffolds due to their broad spectrum of biological activities, including analgesic, anticancer, antidiabetic, and antiviral effects, and their relatively straightforward synthesis, which facilitates their exploration in medicinal chemistry [11, 12]. In vitro biological assays have shown that pyrazolone derivatives display anti‑*T. cruzi* activity comparable to that of benznidazole, including in three-dimensional cardiac microtissue models, highlighting their potential as antichagasic candidates [13, 14].

In this context, the development of compounds that selectively modulate essential molecular targets of *T. cruzi* is particularly attractive. Among these targets, the cysteine protease cruzain (Cz) plays key roles in metacyclogenesis, parasite replication, and host-cell invasion and is expressed across all major parasite life-cycle stages, making it a validated and selective molecular target for antichagasic drug discovery [15, 16]. Despite advances in CD drug research, translating Cz inhibitors into effective new therapies has been slow and remains challenging [17].

Drug discovery is inherently time-consuming, costly, and failure-prone, with overall development often requiring more than a decade and substantial financial investment [18]. These limitations have motivated the widespread adoption of computer-aided drug design (CADD) strategies to prioritize the most promising candidates for biological evaluation, using methods such as molecular docking and quantitative structure–activity relationship (QSAR) modeling to correlate molecular features with biological activity and to screen large libraries of compounds [18–20]. However, more robust physics-based approaches, including all-atom molecular dynamics (MD) simulations and free-energy calculation methods (e.g., end-points and alchemical techniques), provide complementary insight by explicitly accounting for protein and ligand flexibility, solvent effects, and binding thermodynamics, thereby refining docking poses and QSAR hypotheses and increasing confidence in the predicted structure–activity relationships [19, 21].

Phenyl-dihydropyrazolone derivatives have already demonstrated antitrypanosomal activity in phenotypic whole-cell assays against intracellular amastigotes, but the molecular determinants of their interaction with Cz remain poorly characterized, limiting the optimization of this compound class [22]. Nonetheless, several lines of evidence support Cz as a plausible primary target for pyrazole-class compounds. Fragment-based analysis of public databases indicates that the pyrazole scaffold is highly enriched among submicromolar-to-low-micromolar cruzipain inhibitors [23]; biochemical assays have demonstrated that pyrazole–imidazoline derivatives directly inhibit *T. cruzi* cysteine protease activity, with docking studies placing them within the enzyme active site [23]; and subsequent structure-based optimization aimed at enhancing Cz affinity has yielded derivatives with intracellular amastigote IC_50_ values approaching those of benznidazole [24]. On this basis, the present study adopts Cz as the primary molecular target for structure-based investigation of phenyl-dihydropyrazolone derivatives [22] while acknowledging that the pIC_50_ values used for QSAR modeling derive from whole-cell antitrypanosomal assays and that additional targets cannot be excluded.

In this work, we integrated ligand-based and structure-based CADD approaches to characterize phenyl-dihydropyrazolone derivatives with reported antitrypanosomal activity and to investigate their putative interaction with Cz as a candidate molecular target. We first employed QSAR modeling and in silico prediction of absorption, distribution, metabolism, excretion, and toxicity (ADMET) properties to prioritize derivatives with favorable activity and drug‑likeness profiles. We then combined molecular docking with all-atom MD simulations and end-point free-energy calculations to refine predicted Cz-bound poses, probe the stability of ligand–enzyme complexes, and estimate relative endpoint binding free-energy profiles for the predicted complexes [25–27]. Together, these complementary methods support pyrazolones as promising antitrypanosomal scaffolds for the development of new trypanocidal agents and provide a computation-driven framework for prioritizing candidates for future biochemical and cellular validation.

### Computational methods

#### 2D-QSAR validation model

The initial dataset comprised 35 phenyl-dihydropyrazolone compounds reported by Sijm et al. [22] (Table S1). Experimental biological activity data were converted to pIC_50_ (− log(IC_50_)), where IC_50_ represents the concentration required to inhibit 50% of intracellular amastigote growth. During QSAR model diagnosis, CP3 and CP132 were identified as outliers based on leverage criteria and were removed before construction of the final regression model. Therefore, the final 2D-QSAR model was built using 33 compounds, which were divided into training and test sets. These pIC_50_ values served as the dependent variable in the QSAR model. CP3 was not included in the final QSAR regression model but was retained in the downstream docking, ADMET, MD, and MM/GBSA analyses because it was the most active compound in the original whole-cell phenotypic dataset, with an experimental pIC_50_ of 6.40. Two-dimensional structures of the derivatives were built in MarvinSketch, and their geometries were optimized in MOPAC2016 [28] using the semiempirical PM6 method [29]. Molecular descriptors were initially calculated in PaDEL [30] and used to divide the dataset into a training (70%) and a test (30%) set with the DataSet Division software, following a diversity-based partitioning strategy recommended for QSAR studies [31]. Additional descriptors were then generated using the ChemDes [32] and UseGalaxy [33] online platforms for QSAR model development. Before modeling, all descriptors were auto-scaled so that each variable had comparable weight in the analysis, in line with current QSAR best practices [34]. Descriptors showing (i) constant behavior (standard deviation < 0.001) or (ii) at least one missing value were removed. Next, data analysis was performed in QSAR modeling software. In this step, descriptors with low linear correlation with biological activity (|*r*|< 0.3 relative to pIC_50_) were discarded. The reduced matrix was then submitted to the ordered predictors selection (OPS) procedure, which ranks descriptors according to their relevance based on partial least squares (PLS) informative vectors. A second variable-selection step was performed using a genetic algorithm implemented in QSARINS 2.2.4 [35], reflecting modern workflows that combine multiple selection strategies before final model construction [36]. Overall, these procedures aimed to retain only descriptors that are informative for modeling the PLS regression between structure and antichagasic activity.

The purpose of QSAR validation is to demonstrate the model’s predictive ability for new compounds, using suitable internal and external statistical criteria [34, 37]. Internal validation was used to assess model stability and reliability, whereas external validation was employed to confirm its generalizability to unseen data [37, 38]. For internal validation, we calculated the coefficient of determination (*R*^2^), the *F*-test (95% confidence, *α* = 0.05), the root mean square error of calibration (RMSEC), the root mean square error of cross-validation (RMSECV), and the leave-one-out cross-validated correlation coefficient (*Q*^2^_LOO_), in agreement with recommended practices [34, 37]. Model robustness was further examined using leave-N-out (LNO) cross-validation in the QSAR modeling package [39], in which different subsets of compounds are omitted during model building [34]. The statistical significance of the regression was evaluated using the *F*-test, *F*(*k*, *n* − *k* − 1; *α* = 0.05), where *n* is the number of compounds and *k* the number of latent variables; higher *F* values indicate more significant models. To rule out chance correlations, *y*-randomization tests of the pIC_50_ values were performed in QSAR modeling, as recommended for robust QSAR development [34]. Finally, leverage versus studentized residual plots were generated to identify potential outliers and define the model’s applicability domain, consistent with current validation principles [37].

External validation, considered essential in QSAR model assessment, was performed using only test set compounds that were not used in model construction [31, 37]. Predictive performance was quantified by the external determination coefficient (*R*^2^_pred_), the Golbraikh–Tropsha slopes *k* (predicted vs. observed) and *k*′ (observed vs. predicted), the coefficients of determination for regressions constrained through the origin (*r*_0_^2^ and *r*_0_′^2^), the concordance correlation coefficient (CCC), the mean absolute error (MAE), the standard error of prediction (SEP), the average relative prediction error (ARE_pred_), and the external prediction parameters *Q*^2^_*F*1_, *Q*^2^_*F*2_, and *Q*^2^_*F*3_. The use of multiple complementary external metrics is consistent with current recommendations for more reliable characterization of QSAR predictivity [34, 38].

#### Molecular docking

The crystallographic structure of Cz complexed with the ligand B95 was retrieved from the Protein Data Bank under the code 3KKU [40], with a resolution of 1.28 Å. Structure preparation was performed using Chimera 1.12 [41], including removal of water molecules, the crystallographic ligand, and non-amino acid residues, followed by addition of hydrogen atoms. Prior to docking, the protonation states of ionizable residues were evaluated using PROPKA 3.1 [42] at pH 7.0. The catalytic dyad residues, Cys25 and His162, were treated with neutral protonation states during docking.

The docking protocol used in the present study was based on our previously validated Cz redocking workflow reported by Barbosa et al. [27]. In that study, the crystallographic ligand B95 from the Cz structure 3KKU was redocked using GOLD 2022.1.0 [43], FITTED [44], and AutoDock Vina 1.2.3 [45]. Protocol reliability was assessed by calculating the heavy-atom RMSD between the redocked and crystallographic B95 poses using fconv 1.24 [46]. Because the same target structure, crystallographic ligand, and docking workflow were adopted in the present work, those previously reported redocking results were used as methodological support for selecting the production docking protocol.

In GOLD, the GoldScore scoring function was applied at 100% search efficiency within a 10 Å radius sphere centered on the crystallographic ligand (*x* =  − 4.385, *y* =  − 37.389, *z* = 3.546 Å). In AutoDock Vina, a cubic grid box was centered on the crystallographic ligand coordinates, and selected active-site residues (Cys25, Trp26, Gly66, Leu67, Gln159, Leu160, and Asp161) were treated as flexible. In FITTED, receptor flexibility was incorporated through an ensemble-based protocol using four Cz crystal structures: 3KKU, 1ME3, 1ME4, and 1AIM. The structures 1AIM, 1ME3, and 1ME4 were aligned to 3KKU, which was used as the reference structure.

Based on this previous redocking validation, FITTED was selected for the production docking calculations because it reproduced the crystallographic binding mode of B95 with an RMSD of 1.017 Å, below the commonly accepted 2.0 Å threshold, and allowed receptor flexibility to be considered through multiple Cz conformations. Therefore, the validated FITTED protocol was subsequently applied to dock CP3, CP98, and the proposed pyrazolone derivatives LMM1–LMM4 into the Cz active site. The top-ranked FITTED poses were used for protein–ligand interaction analysis and as initial structures for molecular dynamics simulations. Hydrogen-bond and hydrophobic interactions were analyzed using the PoseView web server [47]. This protocol has been successfully applied previously [27].

#### ADMET analysis

In silico tools for predicting ADMET properties enable the determination of pharmacokinetic and toxicological effects of drug candidates prior to the later stages of drug development [20]. Three distinct computational tools were employed to calculate ADMET properties from SMILES structures. Toxicity risks of the studied compounds, including mutagenicity, tumorigenicity, irritant effects, and effects on the reproductive system, were assessed using the OSIRIS Property Explorer program [48]. In the program, the molar mass, solubility, partition coefficient, *Drug-Likeness* (DL), and *Drug-Score* (DS) were calculated. Using SwissADME [49], a free web-based tool, we evaluated the drug-likeness and key pharmacokinetic parameters of the studied compounds. We also assessed whether these substances satisfied Lipinski’s rule of five [50], Ghose [51], Veber [52], Egan [53], and Muegge [54] rules and determined their synthetic accessibility (SA) scores. On the ADMET-AI machine learning platform [55], the structures were submitted for evaluation of intestinal permeability in Caco-2 cells, mutagenicity by the Ames test, acute toxicity (median lethal dose, LD_50_), and drug-likeness using the quantitative estimate of drug-likeness (QED).

#### Molecular dynamics (MD) simulations and structural analysis

Partial atomic charges for all ligands were derived at the Hartree–Fock level of theory with the 6-31G* basis set [56], followed by RESP fitting [57] implemented in Gaussian 09 [58]. In the MD simulations, the catalytic dyad was kept in the neutral Cys25/His162 protonation state, consistent with the docking protocol. His162 was modeled as HIE, based on the hydrogen-bonding pattern observed in the prepared structure and PROPKA 3.1 [42] results. All ligands were parameterized using GAFF [59], and the protein was described using the ff14SB force field [60]. Each Cz complex was inserted into an octahedral solvation box containing TIP3P water molecules [61], with a minimum distance of 10 Å between any solute atom and the box edge. According to the protonation states assigned at pH 7.0, the protein–ligand complexes had a net charge of − 13 e. Therefore, 13 Na⁺ counterions were added solely to neutralize the total system charge. No additional NaCl was added; thus, the explicit-solvent MD simulations were performed with neutralizing counterions only and do not reproduce physiological ionic strength. All systems were built in the tLeap module of AMBER20 [62].

The energy minimization protocol was implemented in four sequential stages, each with 10,000 steps. The initial stage used 7500 steps with the steepest descent method, followed by 2500 steps with the conjugate gradient algorithm. The systems were gradually heated from 100 to 310 K in the NVT ensemble, using Langevin dynamics [63] with a restraint constant of 50 kcal mol^−1^ Å^−2^ applied to heavy atoms. Following a density equilibration protocol comprising six sequential stages, each lasting 200 ps in the NPT ensemble, the restraint constant was gradually decreased until fully released. Long-range electrostatic interactions were calculated using the PME method [64], with a 10 Å non-bonded cutoff. Throughout all NPT simulation stages, covalent bonds involving hydrogen atoms were constrained using the SHAKE algorithm [65], allowing an integration timestep of 2.0 fs with the Verlet algorithm [66]. Finally, the production phase was executed in triplicate, with each replica corresponding to 250 ns of simulation in the NPT ensemble at 310 K and 1 atm. This approach yielded a total sampling time of 750 ns per system or 4500 ns across all six ligand-bound systems. For each system, the three independent replicas were initiated from the same minimized protein–ligand complex but with distinct randomized initial velocities. All MD simulations were carried out using the PMEMD GPU module [67] of AMBER20. Trajectory analyses were performed using the CPPTRAJ program [68], including calculations of RMSD (root mean square deviation), RMSF (root mean square fluctuation), PCA (principal component analysis) [69], and FEL (free energy landscape) [70]. PCA was performed by concatenating trajectories from all six systems following protein Cα alignment, after which a single global covariance matrix was diagonalized. The resulting global principal components served as common axes for projecting the conformational sampling of each individual complex. Consequently, the per-system PC1 and PC2 values reported here represent variance projected along the global principal component axes, rather than independently ranked eigenvalues from separate PCA calculations for each system.

The sensitivity of apo Cz dynamics to the protein force-field and water-model combination was evaluated by conducting additional 500 ns apo simulations using ff14SB/TIP3P and ff19SB/OPC [71] under equivalent simulation conditions. Protein Cα RMSD and radius of gyration were calculated to compare global backbone stability and compactness between the two setups. The corresponding plots are available in the Supporting Information (Fig S1).

#### Binding free energy calculations

Following structural characterization by MD, we calculated the binding free energy (Δ*G*_bind_) using the molecular mechanics/generalized born surface area (MM/GBSA) approach [72, 73], implemented in the MMPBSA.py program [74]. For each system, the last 20 ns of each of the three simulation replicas was utilized, totaling 60 ns of sampling per system. The binding free energy was calculated according to the following thermodynamic relationship:

$$\triangle G_{bind}=\triangle H-T\triangle S\approx\triangle E_{MM}+\triangle G_{solv}-T\triangle S$$

where the enthalpy term (Δ*H*) is approximated by the sum of the molecular mechanics energy (Δ*E*_MM_) and the solvation free energy (Δ*G*_solv_). The Δ*E*_MM_ component encompasses contributions from internal energy (Δ*E*_int_), electrostatic (Δ*E*_elec_), and van der Waals (Δ*E*_vdW_) terms. The solvation energy (Δ*G*_solv_) results from the combination of the polar electrostatic contribution (Δ*G*_GB_), calculated by the Generalized Born model, and the non-polar contribution (Δ*G*_np_), estimated through the product of surface tension (γ) and solvent-accessible surface area (SASA). The entropic term (*T*Δ*S*) was omitted from the calculations due to its high computational cost and associated uncertainties [72], since our primary objective was to compare structurally related compounds under similar conditions.

Additionally, per-residue energy decomposition was performed to estimate individual energetic contributions of selected amino acids in the Cz active site. This analysis used the same time interval as the MM/GBSA calculations, namely, the last 20 ns of each replica. Because ligand RMSD analysis indicated compound-dependent rearrangements relative to the initial docked poses, the decomposition results were interpreted as qualitative energetic descriptions of MD-relaxed binding modes rather than as direct energetic validation of the original docking poses.

## Results and discussion

### 2D-QSAR model: construction, validation, and molecular design

To rationally explore the antichagasic potential of phenyl‑dihydropyrazolones, we first analyzed their experimental whole‑cell activity data and built a two-dimensional QSAR (2D‑QSAR) model. This required transforming the published IC_50_ values reported by Sijm et al. [22] for a focused set of pyrazolone derivatives into a more convenient logarithmic scale and assessing the distribution of potencies across the series as a basis for subsequent modeling.

**Fig. 1:**
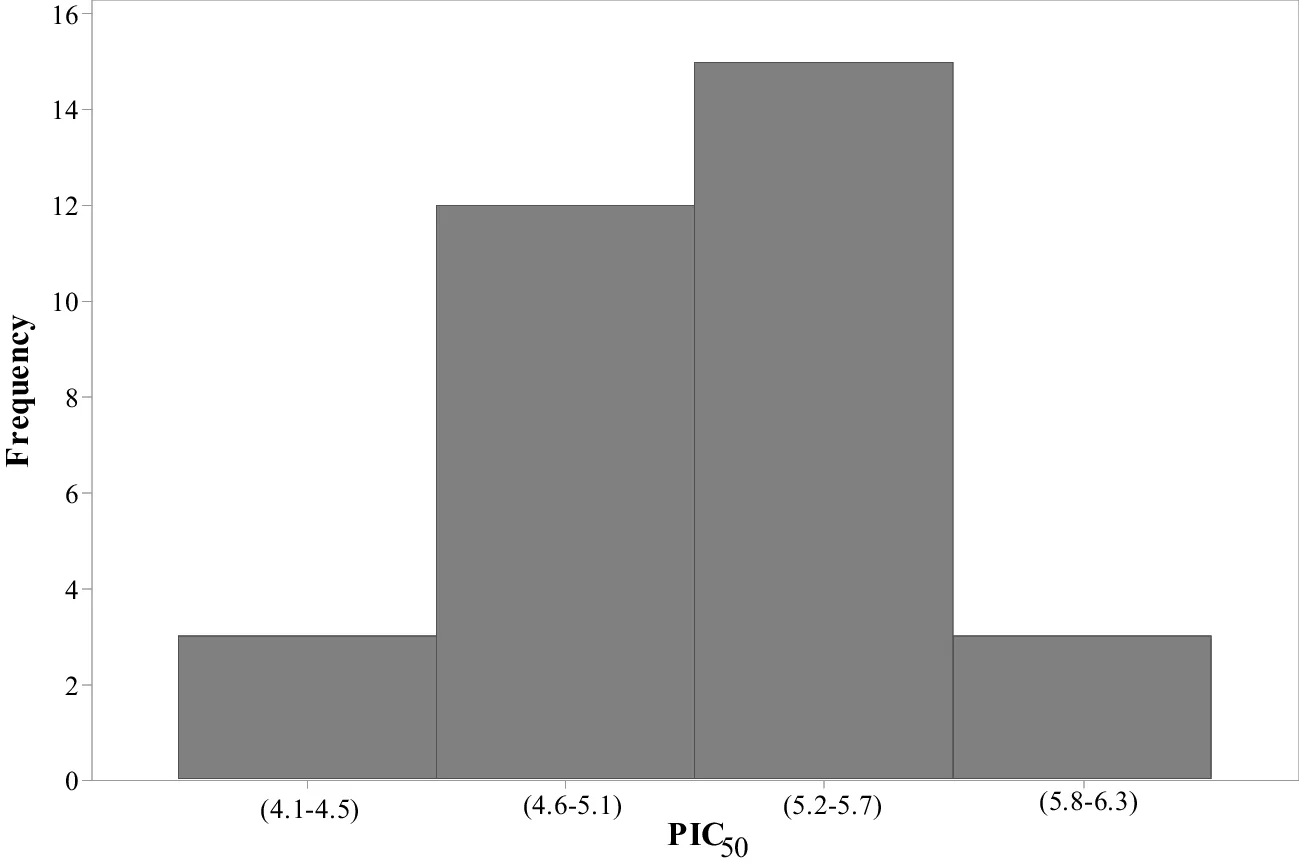
Distribution of pIC_50_ values for the final 33 phenyl-dihydropyrazolone derivatives retained for QSAR model development after outlier removal. Values range from 4.1 to 6.30, with a mean of 5.23, demonstrating adequate variance for QSAR modeling

In the experimental study by Sijm et al. [22], the IC_50_ values (the concentration required to inhibit 50% of parasitic activity) were determined for an initial set of 35 pyrazolone derivatives evaluated in whole-cell assays against intracellular amastigotes of *T. cruzi*. These antichagasic activity values were converted to pIC_50_ (− log(IC_50_)). During QSAR model diagnosis, CP3 and CP132 were identified as outliers based on leverage criteria and were removed from the final regression model. Thus, the final QSAR dataset comprised 33 compounds, with pIC_50_ values ranging from 4.1 to 6.3 (Fig. 1), indicating a favorable distribution of biological activities. CP3, although excluded from the final QSAR model, was retained in the downstream structure-based analyses because it was the most active compound in the original whole-cell dataset, with an experimental pIC_50_ of 6.40.

Initially, the most stable conformation of each compound retained in the final 33-compound QSAR dataset was obtained using the semiempirical PM6 method [29]. Subsequently, 140 descriptors were calculated using the PaDEL program [30] to separate the training and test sets, employing the DataSet Division tool [75] and the Kennard–Stone algorithm [76]. The QSAR model was built using a training set of 23 compounds (70%) and a test set of 10 compounds (30%), which included CP64, CP65, CP66, CP68, CP89, CP91, CP99, CP104, CP133, and CP135. Subsequently, out of the 4700 descriptors initially calculated, only 342 were selected after processing with the QSAR modeling package [39], which removed those with an absolute correlation (|*r*|) of less than 0.3 with the biological activity (pIC_50_). The descriptor matrix was then subjected to a further variable reduction step using the ordered predictors selection (OPS) algorithm [77], available in the QSAR modeling program. As a result, a final matrix containing twelve descriptors was obtained. Finally, QSARINS 2.2.4 [35] was used to reduce the set to five descriptors employed in the construction of the QSAR model. The PLS regression model in QSAR modeling yielded 3 latent variables (3 LVs), accounting for 73.09% of the accumulated information (Table 1). In the statistical validation analysis, an explanatory capacity of 89.2% and a predictive capacity of 80.5% of the total variance were observed, denoted by *R*^2^ and *Q*^2^, respectively. It is noteworthy that these values exceed the minimum thresholds (*R*^2^ ≥ 0.6 and *Q*^2^ > 0.5) considered satisfactory for a QSAR model [78]. To address the influence of the removed outliers, regression coefficients obtained before and after outlier removal are reported in Table S2, showing that the descriptor signs and overall model interpretation were preserved. These results demonstrate that the model exhibits good explanatory power to describe the variation in biological activity of the molecules used in its construction, as well as good predictive performance for pIC_50_ values.

**Table 1:** Validation parameters for the constructed 2-D QSAR model

Statistical parameters	Value
3 LVs	73.09%
*k*	5
*n*	23
*R*^2^	0.892
*Q*^2^_LOO_	0.805
*R*^2^-*Q*^2^_LOO_	0.087
PRESS_cal_	0.89
PRESS_val_	1.612
SS_y_	8.278
PRESS_cal_/SS_y_	0.108
PRESS_val_/SS_y_	0.195
RMSE_val_	0.265
RMSE_cal_	0.197
*R*^2^_adjusted_	0.882
*R*^2^-*R*^2^_adjusted_	0.017
*F*	52.31
*F*	3.13
*F*/*F*	16.71

*LVs* latent variable, *k* number of descriptors in the model QSAR, *n* number of compounds, *R*^*2*^ coefficient of determination, *Q*^*2*^_*LOO*_ cross-validation coefficient of determination, *PRESS*_*cal*_ predictive residual sum of squares of calibration, PRESS_val_ predictive residual sum of squares of validation, *SS*_*y*_ sum of squares of the response values, *RMSE*_*val*_ root mean square error of validation, *RMSE*_*cal*_ root mean square error of calibration, *R*^*2*^_*adjusted*_ adjusted correlation coefficient, *F*_*(k,n-k-1)calc*_ calculated *F* test, *F*_*(k, n-k-1)table*_ reference value *F* test

Analyzing the *R*^2^ and *Q*^2^_LOO_ values, it was found that the difference between them was 0.087, which is within the limit suggested by Kiralj and Ferreira, indicating that the model has a low probability of data overfitting [78]. The predictive residual sum of squares (PRESS_cal_) indicates that smaller discrepancies between experimental and predicted biological activity values correspond to greater predictive capacity of the linear model [25]. As shown in Table 1, both PRESS_cal_ and PRESS_val_ are lower than the total sum of squares of the response values (SSy), demonstrating that the model was not obtained by chance and can therefore be considered statistically significant [79]. Furthermore, the PRESS/SSy ratio is below 0.4, indicating good predictive capacity for the proposed model [80]. The predictive utility of the QSAR model for pyrazolone-derived compounds is further supported by the root-mean-square errors of calibration and validation (RMSE_cal_ and RMSE_val_), which show only slight deviations between predicted and experimental values. As shown in Table 1, the calculated *F*-statistic (95% confidence level, *α* = 0.05) exceeds the critical tabulated value, indicating a statistically significant model in which the selected descriptors genuinely predict pIC_50_ values [81]. Additionally, the adjusted coefficient of determination (*R*^2^_adjusted_), which accounts for the number of compounds and latent variables in the PLS-derived model, indicates that the regression equation has substantial explanatory power. In addition, the model includes an acceptable number of descriptors (*n* = 5), as indicated by the difference between *R*^2^ and R^2^_adjusted_ remaining below 0.3 [82]. The selected descriptors and corresponding biological activity (pIC_50_) values for the compounds used to construct the QSAR model are summarized in Table 2. All five descriptors (GATS8c, RDFC24, TPSA, MoRSEE28, and PEOEVSA7) were obtained from the descriptor pool calculated using ChemDes [32] and UseGalaxy [33], as described in the Computational methods section.

**Table 2:** Selected descriptors for the 2-D QSAR model and pIC_50_ values

Compound	GATS8c	RDFC24	TPSA (Å^2^)	MoRSEE28	PEOEVSA7	pIC_50_^[c]^
CP57ª	1.033	0.005	54.79	0.066	29.562	5.1
CP58ª	1.137	0.037	64.02	− 0.082	35.126	4.3
CP59ª	1.112	− 0.042	54.79	0.044	31.109	5.8
CP60ª	1.277	0.000	54.79	0.034	35.126	5.1
CP61ª	0.917	− 0.011	45.56	− 0.047	23.999	5.5
CP62ª	0.984	− 0.004	45.56	− 0.013	29.562	5.2
CP63ª	0.905	− 0.004	45.56	− 0.166	34.585	5.5
CP64^b^	1.052	− 0.002	45.56	− 0.077	23.999	5.6
CP65^b^	1.277	− 0.016	54.79	0.184	29.562	5.6
CP66^b^	1.318	− 0.025	54.79	0.400	41.192	5.3
CP67ª	1.128	− 0.020	54.79	0.173	29.562	4.9
CP68^b^	1.236	0.004	54.79	− 0.090	35.126	4.5
CP74ª	1.012	− 0.012	69.35	− 0.138	29.562	4.3
CP89^b^	0.986	0.019	54.79	− 0.179	29.562	5.2
CP90ª	1.057	− 0.007	54.79	− 0.296	29.562	5.1
CP91^b^	1.081	0.040	58.03	0.053	29.562	4.5
CP92ª	1.167	0.042	64.02	0.212	42.776	4.5
CP95ª	0.720	0.047	49.17	0.511	35.150	4.5
CP98ª	0.821	0.006	73.92	0.323	29.941	4.1
CP99^b^	1.454	− 0.004	37.72	0.426	29.562	6.3
CP104^b^	1.138	0.000	54.79	0.322	42.404	5.0
CP109ª	1.230	0.025	65.19	0.132	29.438	4.8
CP120ª	1.032	0.000	43.38	0.314	29.562	5.7
CP121ª	1.150	0.012	43.38	0.600	23.521	6.0
CP122ª	1.082	0.022	43.38	0.572	23.521	5.9
CP123ª	1.129	0.000	43.38	0.564	23.521	6.2
CP124ª	1.142	0.024	43.38	0.324	23.521	5.7
CP133^b^	1.115	0.002	55.41	− 0.031	23.521	5.0
CP134ª	1.108	0.019	64.64	0.116	36.734	4.6
CP135^b^	1.148	0.004	55.41	0.389	23.521	5.8
CP136ª	1.084	0.013	55.41	0.470	23.521	5.7
CP141ª	1.053	0.012	34.15	0.221	35.315	5.6
CP142^a^	1.072	0.026	34.15	0.419	35.315	5.7

^a^Training set; ^b^test set; ^[c]^phenotypic activity of pyrazolone compounds against intracellular amastigotes of *T. cruzi* (Tulahuen strain)

Equation (1) represents the best regression model obtained with five descriptors for the training set:

1 $$pIC_{50pred}=1.281\left(GATS8c\right)-7.463\left(RDFC24\right)-0.039\left(TPSA\right)+0.449\left(MoRSEE28\right)-0.027\left(PEOEVSA7\right)+6.7$$

Equation (1) shows that the predicted pIC_50_ values are derived from a linear combination of the molecular descriptors selected during QSAR model construction. Therefore, within the multivariate structure of the model, compounds with higher predicted activity are favored by higher values of GATS8c and MoRSEE28 and by lower values of RDFC24, TPSA, and PEOEVSA7. The Geary autocorrelation at lag 8, weighted by atomic charges (GATS8c), quantifies the extent of charge heterogeneity between pairs of atoms separated by eight bonds [83]. Notably, compounds CP99 (test set; pIC_50_ = 6.3; GATS8c = 1.454) and CP123 (training set; pIC_50_ = 6.2; GATS8c = 1.129) were among the most active compounds in the final QSAR dataset and displayed relatively high GATS8c values. This behavior indicates that charge-weighted topological relationships at this molecular length scale are relevant for the QSAR model. However, GATS8c should be interpreted as a statistical descriptor of charge distribution along the molecular graph rather than as a direct measure of π-electron density. The most significant descriptor in Eq. (1) was the radial distribution function based on atomic charge (RDFC24), a charge-weighted radial distribution descriptor that captures how atomic charge information is distributed over interatomic distances [83]. As shown in Table 2, RDFC24 values remain below 0.05. According to Eq. (1), lower values of this descriptor contribute favorably to predicted pIC_50_ values within the multivariate model. The physicochemical descriptor topological polar surface area (TPSA) corresponds to the sum of the surface areas of all polar atoms in a molecule, typically oxygen and nitrogen, including their bonded hydrogen atoms. This descriptor is commonly employed in drug design to predict cell permeability [84]. In the present dataset, several highly active compounds displayed relatively low TPSA values (Table 2). This contrast is evident when CP99, the most potent compound in the test set (TPSA = 37.72 Å^2^), is compared with CP98, the least active compound (TPSA = 73.92 Å^2^), consistent with the negative coefficient obtained for TPSA in Eq. (1) of the PLS model. The 3D MoRSE descriptors (3D Molecule Representation of Structures based on Electron diffraction) are derived from infrared-spectrum simulations using a generalized scattering function [85]. Specifically, the descriptor employed herein is the 3D MoRSE descriptor based on atomic Sanderson electronegativity (MoRSEE28). Table 2 shows that compounds with higher biological activity, including CP121, CP122, CP123, and CP99, showed relatively high MoRSEE28 values. However, CP95 also displayed a high MoRSEE28 value despite its lower activity, indicating that this descriptor should be interpreted as part of a multivariate QSAR trend rather than as a strictly monotonic predictor of activity.

Furthermore, the descriptor Partial Equalization of Orbital Electronegativity of Van der Waals Surface Area descriptor 7 (PEOEVSA7) quantifies atomic charge to represent electrostatic interactions. PEOEVSA7 is calculated by determining the van der Waals surface area (VSA) obtained from the partial charges of atoms using partial equalization of orbital electronegativities (PEOE) [83]. Its negative coefficient in Eq. (1) is consistent with the observation that the most active compounds CP121, CP122, and CP123 displayed comparatively low values of this variable.

In Table 3, the coefficients of Eq. (1) and the Pearson correlation coefficient (*r*) values for each descriptor with pIC_50_ were evaluated. Table 3 shows that the model has descriptors whose coefficient signs match those predicted by the correlation with biological activity, indicating the self-consistency of the proposed QSAR model [78].

**Table 3:** Standardized regression coefficients of the 2-D QSAR model and Pearson correlation coefficient (*r*)

Descriptors	Standardized regression coefficient	*R*
GATS8c	0.264	0.236
RDFC24	− 0.253	− 0.273
TPSA	− 0.690	− 0.807
MoRSEE28	0.188	0.405
PEOEVSA7	− 0.236	− 0.546

**Fig. 2:**
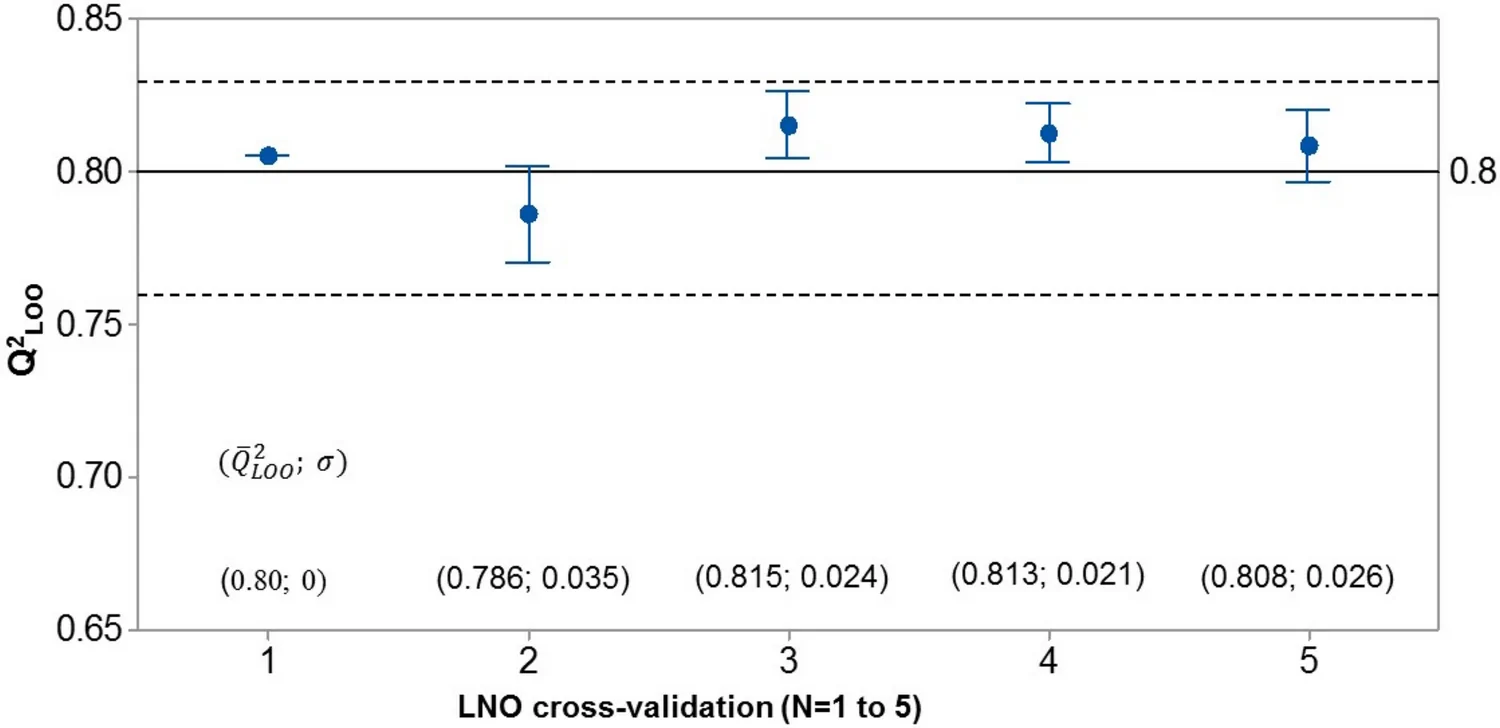
Leave-N-out (LNO) cross-validation plot. Mean *Q*^2^ values (*y*-axis) obtained after removing *N* = 1 to 5 compounds from the training set (*x*-axis), repeated five times per *N* value with row randomization. Error bars represent standard deviations. The dashed line indicates the minimum acceptable threshold (*Q*^2^ = 0.5)

The LNO cross-validation test was repeated five times for each *N* value, with 1 to 5 compounds removed from the training set. For each replicate, the rows of the data matrix were randomized, and the mean *Q*^2^_LOO_ values were calculated (Fig. 2). As can be observed, the mean *Q*^2^ values (${\overline{Q} }_{\mathrm{L}\mathrm{O}\mathrm{O}}^{2},$ leave-2-out to leave-5-out) exceed the threshold recommended in the literature (*Q*^2^ > 0.5), indicating that the model retains its predictive capability even when up to five compounds are excluded [78, 86]. Moreover, these values are comparable to the *Q*^2^_LNO_ obtained with leave-1-out, with the difference between each *Q*^2^_LNO_ and *Q*^2^_LOO_ being less than 0.1 and the standard deviations (σ) remaining below 0.1 for *N* = 2–5, criteria that are commonly taken as evidence of model robustness in LNO analyses [78].

**Fig. 3:**
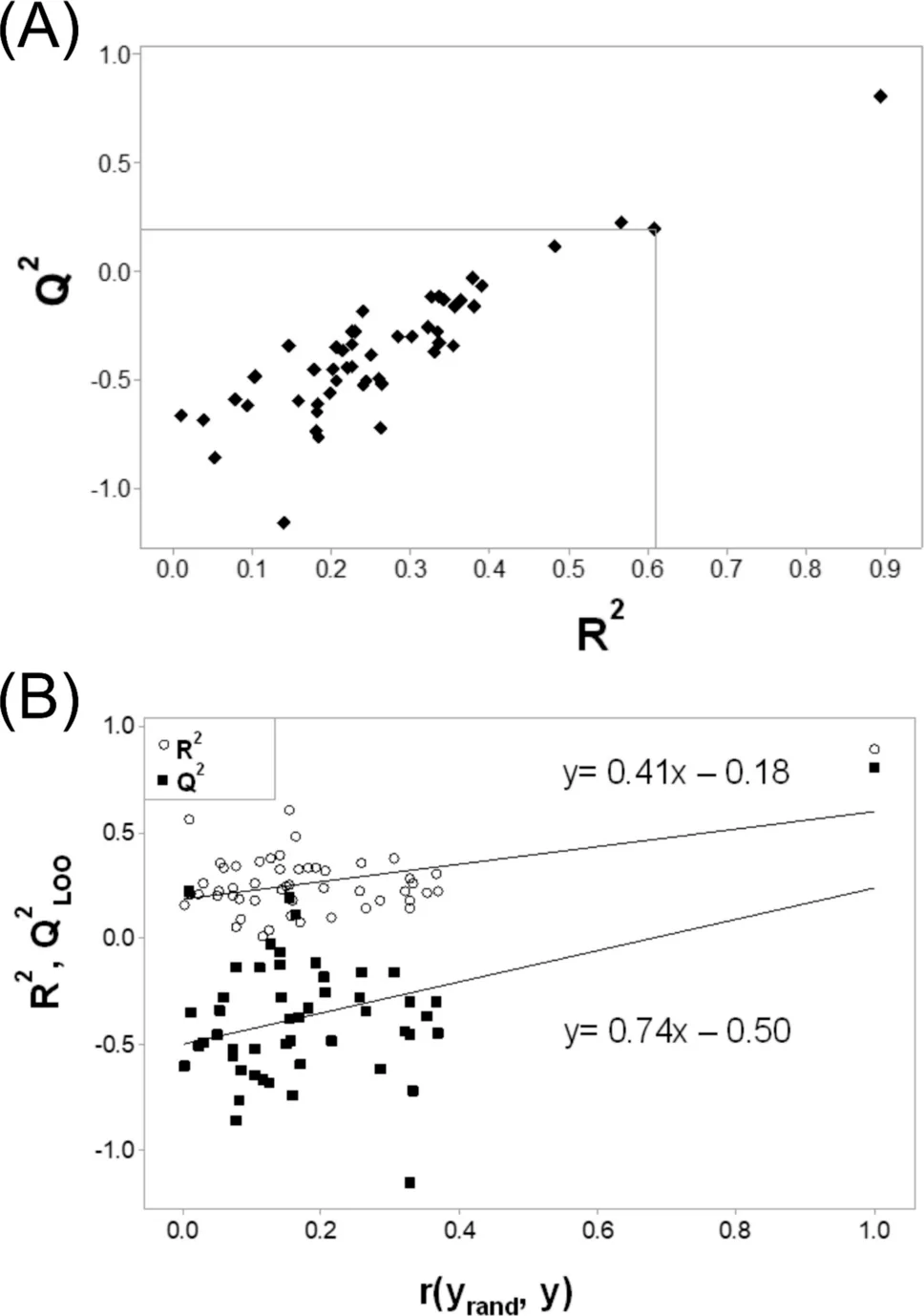
Results of the *y*-randomization test. **A** *Q*^2^ versus *R*^2^ for the randomized models, where *Q*^2^ denotes the coefficient of determination obtained from L_OO_ cross-validation. **B** Linear regression of *r*_(yrand, y)_ against R^2^ (upper line) and Q^2^_L__OO_ (lower line) for the randomized models

The possibility of chance correlation was tested using *y*-randomization analysis, with the results presented in Fig. 3A. In this procedure, the **y** vector (pIC_50_ values) was scrambled 50 times. For each iteration, the original descriptor matrix was kept fixed and the *y* vector was randomized. Thus, the randomized models were expected to perform relatively poorly compared to the original, yielding *R*^2^ values below 0.3 and *Q*^2^ values below 0.05, which are lower than the original metrics [78]. Comparing of the randomized models with the proposed QSAR model, as shown in Fig. 3A, revealed no evidence of chance correlation, as all randomized models exhibited lower predictive (*Q*^2^) and explanatory (*R*^2^) values exhibit lower values, with means of − 0.39 and 0.25, respectively, compared to the actual model, reflecting the credibility and robustness of the constructed QSAR model. From Fig. 3B, it can be observed that the intercepts of the linear regression equations for *r*_(yrand, y__)_ versus *Q*^2^, and *r*_(yrand, y)_ versus *R*^2^ are lower than 0.3 and 0.05, respectively. These findings demonstrate that the variance explained by the model is not due to chance correlation, and therefore the model can be considered robust [78].

The applicability domain was evaluated to assess whether the developed QSAR model yields reliable predictive performance [87]. This concept is essential for identifying structural and response outliers within the training and test sets. The leverage approach was employed to evaluate the chemical space of the final QSAR model using the Williams plot, in which standardized residuals are plotted on the *x*-axis against leverage values on the *y*-axis [88].

**Fig. 4:**
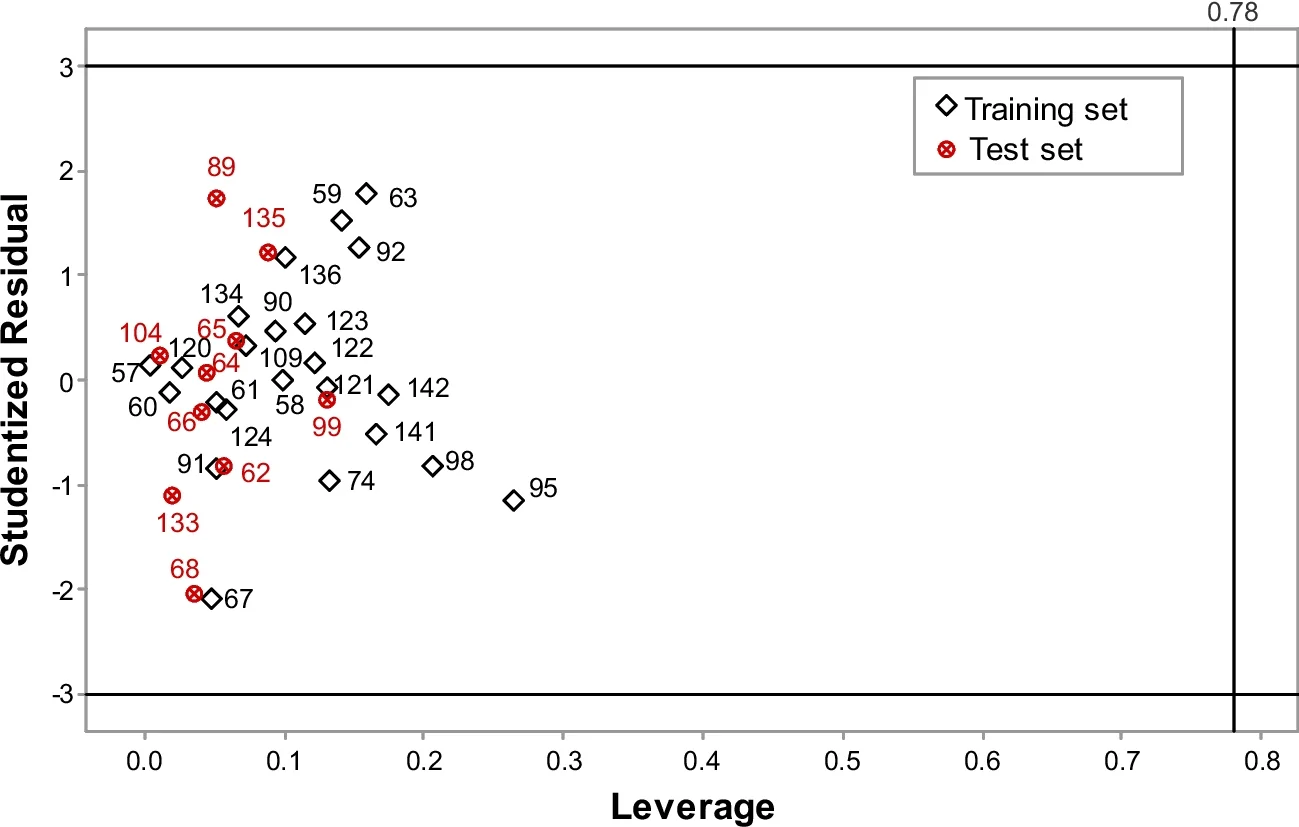
Williams plot showing Studentized residuals plotted against leverage values for the final 33-compound QSAR model after removal of CP3 and CP132. Training-set compounds are shown as filled circles and test-set compounds as open circles. The vertical line indicates the warning leverage threshold (h* = 0.783), and the horizontal lines indicate the ± 3 studentized residual limits. Compound labels are shown using the CP nomenclature

The results of this analysis (Fig. 4) showed that, after removal of CP3 and CP132, all compounds retained in the final training and test sets fell within the applicability domain. Their leverage values were below the warning leverage (h* = 0.783, calculated as h* = 3 × (*k* + 1)/*n*, where *k* = 5 descriptors and *n* = 23 compounds in the training set), and their standardized residuals lie within the ± 3 range [89]. These observations indicate that no additional structural or response outliers remained in the final QSAR dataset, supporting the model’s suitability for exploratory design of novel pyrazolone derivatives within its applicability domain.

External validation of the proposed QSAR model was performed using the test set of 10 pyrazolone derivatives to evaluate its predictive performance on compounds not used for model construction. The results are shown in Table 4, where the values of the first four parameters satisfy the statistical conditions recommended in the literature for an acceptable QSAR model [34, 92]. The statistical parameters confirmed the external predictive reliability of the model. The *Q*^2^_*F*1_, *Q*^2^_F2_, and *Q*^2^_*F*3_ values of 0.978, 0.701, and 0.685, respectively, all surpassed the recommended threshold of 0.5, collectively corroborating the robustness of the external predictions. Furthermore, the concordance correlation coefficient (CCC = 0.839) indicated satisfactory agreement between the predicted and experimental values. Additionally, the low mean absolute error (MAE = 0.23) reflected the small magnitude of prediction deviations across the test set. It is also noteworthy that the standard error of prediction (SEP) and the average relative error (ARE_pred_) are low [38], indicating small deviations between the predicted and experimental pIC_50_ values. The difference between *Q*^2^_*F*1_ and *Q*^2^_*F*2_/*Q*^2^_*F*3_ reflects the sensitivity of external validation metrics to the reference mean and intercept correction in a small test set. Therefore, external predictivity was assessed using the combined set of external metrics rather than relying on a single parameter. Taken together, these results indicate that the proposed QSAR model can be used to estimate the pIC_50_ values of new pyrazolone derivatives within its applicability domain.

**Table 4:** Results of statistical parameters and predicted values obtained for the test set

Parameter	Result	Recommended criterion [31]
*R*^2^_pred_	0.701	> 0.6
*k*	0.982	0.85 < *k* < 1.15
*k*′	1.015	0.85 < *k*´ < 1.15
$\left|{\mathrm{R}}_{0}^{2}-{\mathrm{R}}_{0}^{^{\prime}2}\right|$	0.074	< 0.3
${Q}_{F1}^{2}$	0.978	> 0.5 [90]
${Q}_{F2}^{2}$	0.701	> 0.5 [90]
${Q}_{F3}^{2}$	0.685	> 0.5 [90]
CCC	0.839	> 0.7 [91]
MAE	0.23	
SEP	0.31	
ARE_pred_ (%)	4.55	

*R*^*2*^_*pred*_ external predictive coefficient of determination, *k* *k*′ Golbraikh–Tropsha slopes of the linear regression lines between the observed and the predicted activities in the external validation, *|R*^*2*^_*0*_*–R′20 |* Golbraikh–Tropsha absolute values of the difference of the determination coefficient of the linear relation between the observed and predicted values without an intercept (*R*^2^_0_) and the predicted and observed values without an intercept (*R*′^2^_0_), *Q*^*2*^_*F1*_ predictive squared correlation coefficient calculated relative to the mean of experimental values, *Q*^*2*^_*F2*_ predictive squared correlation coefficient using the mean of predicted values, *Q*^*2*^_*F3*_ predictive squared correlation coefficient adjusted for intercept deviations from the experimental mean, *CCC* concordance correlation coefficient, *SEP* standard error of prediction, *MAE* mean absolute error, ARE_pred_ (%) average relative error of prediction

**Fig. 5:**
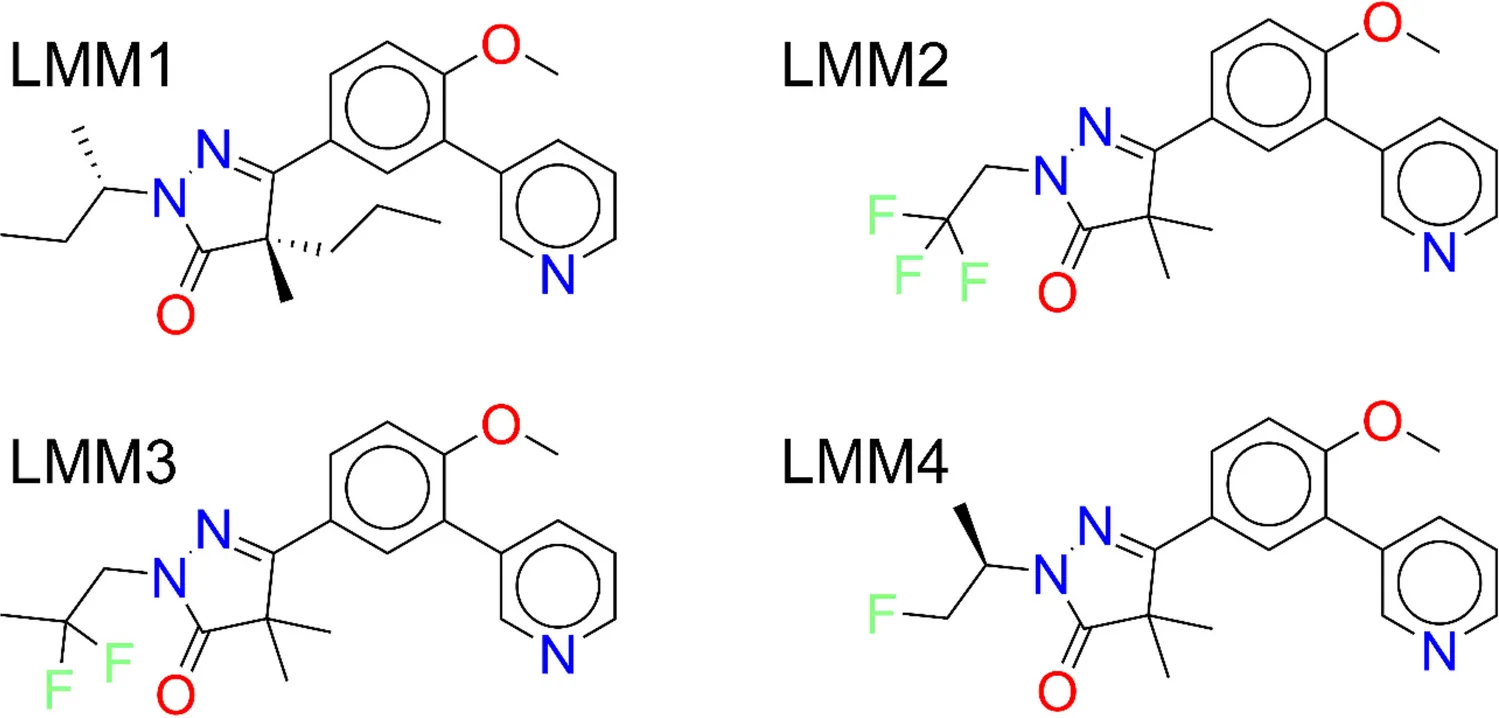
Representative 2D structures of the LMM1-LMM4 proposed on the basis of the 2D-QSAR model developed in this study

Based on the QSAR model’s analysis of biological activity, four new pyrazolone derivatives, designated LMM1–LMM4, were proposed (Fig. 5). Their pIC_50_ values were predicted using the linear model (Eq. 1). As shown in Table 5, these values are intermediate within the activity range of the previously published pyrazolone compounds reported by Sijm et al. [22]. Therefore, LMM1–LMM4 were not proposed because they were predicted to exceed the most potent compounds in the original dataset but because they combine structural novelty, acceptable predicted antitrypanosomal activity, and favorable preliminary drug-likeness/ADMET profiles. Accordingly, the QSAR model developed in this study was used as an exploratory tool to guide the design of new pyrazolone derivatives within its applicability domain.

**Table 5:** Descriptors for the proposed pyrazolone derivatives (LMM1 to LMM4) and biological activity values predicted by the 2-D QSAR model

Compound	GATS8c	RDFC24	TPSA	MoRSEE28	PEOEVSA7	pIC_50pred_
LMM1	0.913	− 0.092	54.79	0.031	23.521	5.8
LMM2	1.219	0.006	54.79	0.165	23.521	5.5
LMM3	1.076	0.003	54.79	0.499	30.444	5.3
LMM4	1.247	0.002	54.79	0.299	23.521	5.6

### Molecular docking calculations

Cz is the major cathepsin L‑like cysteine protease of *T. cruzi* and a validated molecular target for antichagasic drug discovery, as it participates in key processes such as parasite proliferation, differentiation, host‑cell invasion, and immune evasion across different life‑cycle stages [93–95]. Structurally, Cz shares the conserved catalytic triad Cys25–His162–Asn182 typical of papain‑like proteases, but its substrate and inhibitor specificity is largely governed by a series of subsites (S3–S1′) that define the active‑site cleft [96, 97]. Among these, the S2 pocket forms a predominantly hydrophobic cavity shaped by residues such as Gly66, Leu67, Met68, Ala133, and Leu160, which can accommodate both bulky aromatic and basic substituents at the P2 position of ligands. In contrast, residues in S3 and S1′, including Gly65, Gly66, and Asp161, contribute additional hydrophobic and electrostatic contacts that are critical for selectivity over human cathepsins [97, 98]. These well‑characterized subsites have been extensively exploited in structure‑based design and docking‑driven optimization of Cz inhibitors, providing a robust framework for interpreting how new phenyl‑dihydropyrazolone derivatives interact with the enzyme and to rationalize their predicted binding modes and affinities in molecular docking studies [16, 94, 98, 99].

The docking workflow adopted in the present study was supported by the redocking validation previously reported by Barbosa et al. [27] for Cz structure 3KKU and its crystallographic ligand B95. In that validation, GOLD, AutoDock Vina, and FITTED were compared by calculating the heavy-atom RMSD between the redocked and crystallographic B95 poses. The reported RMSD values were 2.827 Å for GOLD, 6.499 Å for AutoDock Vina, and 1.017 Å for FITTED. Based on this previous validation, FITTED was selected for production docking in the present work because it reproduced the crystallographic binding mode within the accepted 2.0 Å threshold and allowed receptor flexibility to be considered through an ensemble-based protocol. Table 6 presents the FITTED RankScore values for CP3, CP98, and the four proposed derivatives LMM1–LMM4. These scores were used for pose selection and qualitative comparison among the docked compounds, rather than as physical binding free energies. The top-ranked FITTED poses were subsequently used for protein–ligand interaction analysis and as initial structures for MD simulations (Fig. S2). Table 7 presents the interactions of the analyzed compounds as visualized by the online server PoseView [100]. Most of the proposed compounds (LMM1–LMM4) formed hydrogen bonds primarily with the amino acid residues Ser61 and Gly66, as well as hydrophobic interactions with residues Gly65, Gly66, and Leu67. The Gly66 residue identified in the docking studies is located in the enzyme’s hydrophobic subsite S3, which plays a key role in Cz selectivity [27]. Subsite S3 of Cz, which includes residues Gly65 and Gly66, exhibits a preference for aromatic or positively charged groups [101]. This feature is crucial for the selectivity of Cz relative to its human homologs, the cathepsins. Among these residues, Ser61 forms hydrogen bonds with inhibitors and constitutes a recognition hotspot in Cz. The importance of this interaction is supported by a study by Cerruti et al. [99], which demonstrated that the absence of Ser61 is a determining factor for the low inhibitory activity observed in certain analogs.

**Table 6:** FITTED RankScore values obtained for CP3, CP98, and the proposed pyrazolone derivatives LMM1–LMM4. Representative 3D conformations are shown in Fig. S2

Compound	FITTED RankScore
CP3	–47.237
CP98	–34.828
LMM1	–49.493
LMM2	–50.361
LMM3	–48.117
LMM4	–46.384

**Table 7:** Hydrogen bonds and hydrophobic interactions with amino acid residues of the Cz enzyme

Compound	Hydrogen bond	Hydrophobic interactions
CP3	No interactions	No interactions
CP98	Asp60, Ser61	Gly65
LMM1	Ser61	Gly65, Leu160
LMM2	Ser61, Ser64, Gly66	Gly65, Gly66, Leu67
LMM3	Ser61	Gly66, Leu67
LMM4	Gly66, Gly208	Gly66, Gly208

### In silico ADMET predictions

ADMET analyses play a critical role in identifying compounds with favorable pharmacokinetic and toxicological profiles, which enhances the clinical success rate of drug candidates in advanced stages of development [102]. The probability of mutagenicity (M), tumorigenicity (T), irritant effects (I), and reproductive toxicity (RE) was assessed using the OSIRIS Property Explorer. The results (Table 8) demonstrated that none of the selected compounds exhibited M, T, I, or RE effects, suggesting a favorable safety profile. The molar mass (MW) of the compounds ranged from 298.36 to 393.52 Da, remaining below the 500 Da threshold, while the cLogP values ranged from 2.23 to 4.86, consistent with conventional drug-likeness criteria. The predicted solubility values ranged from − 5.63 to − 3.92 and were therefore interpreted comparatively rather than as evidence of high aqueous solubility. The drug-likeness (DL) values were predominantly positive, except for LMM2, which had a value of –2.05, indicating that this compound lacks fragments frequently found in commercial drugs [103]. The highest drug-score (DS) values were obtained for the molecules synthesized by Sijm and colleagues, 0.70 and 0.81 for CP3 and CP98, respectively. Notably, compound LMM4 showed a value (0.69) close to that of CP3. The DS is a parameter that synthesizes the overall potential activity of the molecule into a single value, integrating information from cLogP, logS, MW, DL, and toxicity risk [104].

**Table 8:** Toxicity parameters properties predicted using the OSIRIS program

Compound	M	T	I	RE	MW	cLogP	Solubility	DL	DS
CP3	NRP	NRP	NRP	NRP	337.42	3.04	− 4.55	3.58	0.70
CP98	NRP	NRP	NRP	NRP	298.36	2.23	− 3.92	4.31	0.81
LMM1	NRP	NRP	NRP	NRP	393.52	4.86	− 5.63	1.56	0.42
LMM2	NRP	NRP	NRP	NRP	377.32	3.17	− 4.91	− 2.05	0.36
LMM3	NRP	NRP	NRP	NRP	373.40	3.39	− 4.89	0.53	0.52
LMM4	NRP	NRP	NRP	NRP	355.41	2.82	− 4.62	3.67	0.69

*NRP* no risk predicted, *M* mutagenicity, *T* tumorigenicity, *I* irritant effect, *RE* reproductive effect, *MW* molecular weight, *cLogP* octanol–water partition coefficient, *DL* drug-likeness, *DS* drug-score

In SwissADME, the drug-likeness of the compounds was assessed using Lipinski’s Rule of Five (hydrogen bond donors (HBD) ≤ 5, hydrogen bond acceptors (HBA) ≤ 10, molecular weight (MW) ≤ 500 Da, and octanol–water partition coefficient (Log P) ≤ 5), the Ghose filter (MW between 40 and 130 Da and total number of atoms between 20 and 70), Veber’s rule (number of rotatable bonds ≤ 10 and polar surface area (PSA) ≤ 140 Å^2^), Egan’s rule (PSA ≤ 131.6 Å^2^ and Log *P* < 5.88), and the Muegge rule (200 ≤ MW ≤ 600 Da, − 2 ≤ Log *P* ≤ 5, total polar surface area (TPSA) ≤ 150 Å^2^, number of aromatic rings ≤ 7, number of rotatable bonds ≤ 15, HBD ≤ 5, HBA ≤ 10, number of carbon atoms > 4, and number of heteroatoms > 1) [105]. All six compounds displayed favorable chemical profiles consistent with high oral bioavailability, thereby fulfilling the drug-likeness criteria proposed by Lipinski, Ghose, Veber, Egan, and Muegge [106]. Furthermore, synthetic accessibility (SA) provides a metric for estimating the ease of synthesis of a molecule, with values ranging from 1 (easily synthesized) to 10 (difficult to synthesize) [107]. Regarding SA, compound CP98 exhibited the lowest SA score (2.98), indicating the greatest predicted synthetic accessibility, whereas LMM1 showed the highest SA score (4.43), indicating the greatest predicted synthetic difficulty. On the other hand, compound LMM1 (4.43) presented the lowest synthetic accessibility, indicating the greatest difficulty of synthesis. Table 9 presents the values calculated using the ADMET-AI platform for Caco-2 permeability, Ames mutagenicity probability, quantitative estimate of drug-likeness (QED), and predicted acute toxicity (LD50) [55]. The Caco-2 and LD50 outputs are reported as ADMET-AI percentiles, allowing comparison with approved drugs catalogued in DrugBank [55]. Caco-2 permeability is commonly used as an in vitro model-related parameter for estimating intestinal drug absorption [103]. The ADMET-AI Caco-2 permeability percentiles ranged from 54.63% to 88.13%, with most compounds showing favorable predicted permeability profiles, particularly CP3 (88.13%) and LMM4 (86.16%). The Ames test is a bacterial mutagenicity assay used to detect compounds with potential to induce genetic damage [103]. The Ames mutagenicity probabilities ranged from 0.30 to 0.50, with LMM4 displaying the highest predicted probability. These values are consistent with the OSIRIS predictions, which classified all compounds as having no predicted mutagenicity risk. Regarding drug-likeness, QED is a quantitative index ranging from 0 to 1 that summarizes the drug-like character of a molecule [108]. Five compounds (CP3, CP98, LMM2, LMM3, and LMM4) showed QED values equal to or above the 0.67 threshold [109], whereas LMM1 presented a slightly lower QED value of 0.61. LD50 represents the estimated dose required to cause lethality in 50% of a test population and is commonly used as an indicator of acute toxicity [110]. The ADMET-AI LD50 percentiles ranged from 53.9% for CP98 to 92.4% for LMM2, suggesting that most compounds fall within a favorable predicted acute-toxicity range relative to DrugBank compounds. Overall, the ADMET-AI predictions support the favorable drug-likeness and preliminary safety profile of the evaluated pyrazolone derivatives.

**Table 9:** Parameters obtained from the ADMET-AI server

Compound	Caco-2 permeability percentile (%)	Ames mutagenicity probability	QED	${\mathbf{L}\mathbf{D}}_{50}$ percentile (%)
CP3	88.13	0.32	0.85	65.2
CP98	54.63	0.39	0.80	53.9
LMM1	70.03	0.3	0.61	68.7
LMM2	81.78	0.41	0.81	92.4
LMM3	82.98	0.44	0.80	89.3
LMM4	86.16	0.5	0.82	89.7

*Caco-2 permeability percentile* ADMET-AI percentile for predicted intestinal permeability relative to DrugBank compounds, *Ames mutagenicity probability* predicted probability of a positive Ames mutagenicity outcome, *QED* quantitative estimate of drug-likeness, *LD50 percentile* ADMET-AI percentile for predicted acute toxicity relative to DrugBank compounds

### Molecular dynamics simulations: structural and conformational analyses

**Fig. 6:**
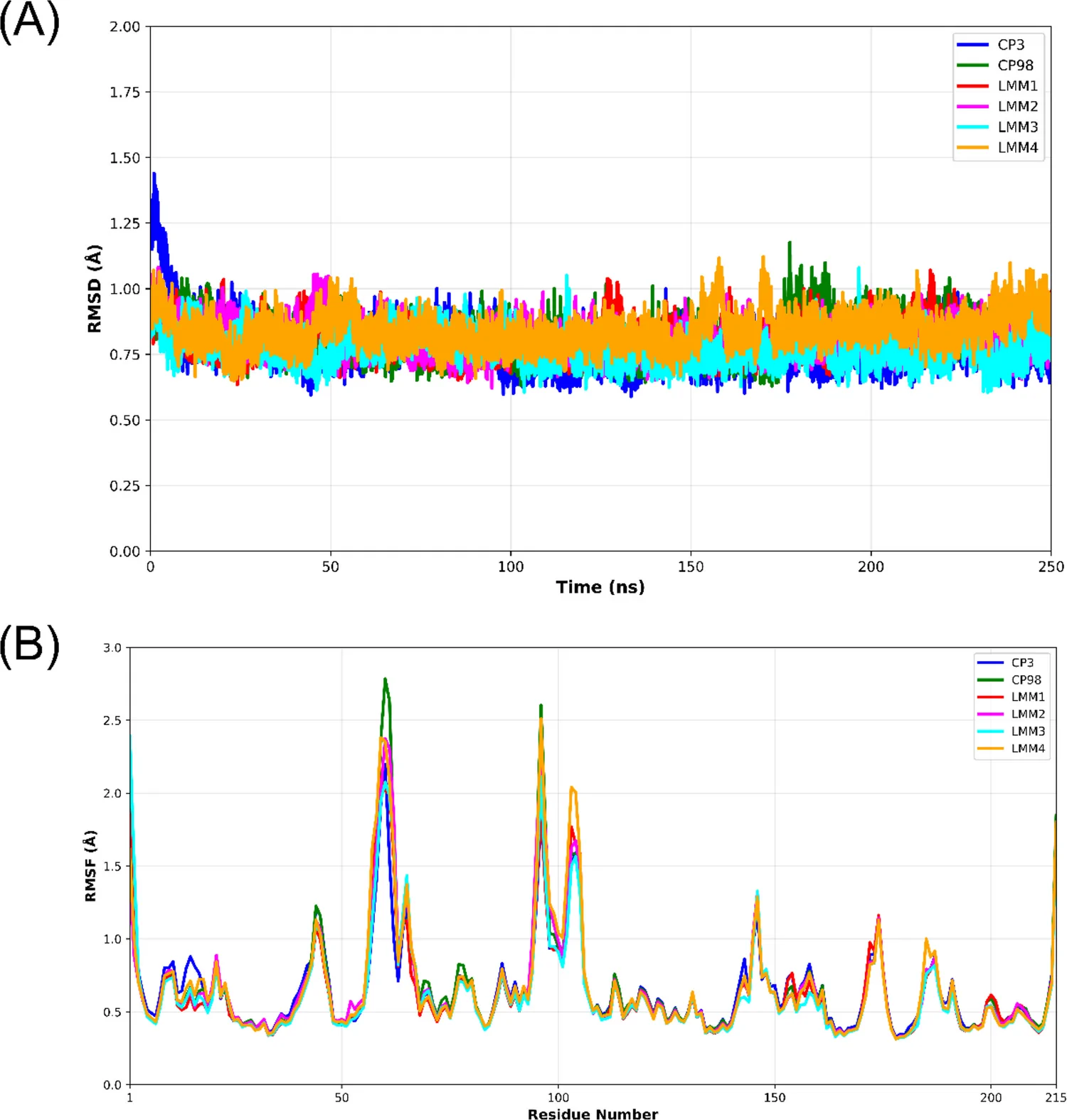
**A** Protein Cα root mean square deviation (RMSD) and **B** protein Cα root mean square fluctuation (RMSF). Data were computed from three independent replicas of 250 ns for each system, corresponding to 750 ns of aggregate sampling per complex

As described in the Computational methods section, MD simulations were performed in triplicate (3 × 250 ns) to evaluate the conformational stability of the six Cz complexes (CP3, CP98, LMM1, LMM2, LMM3, and LMM4). For each system, the replicas were initiated from the same minimized protein–ligand complex using different randomized initial velocities. A similar ff14SB/TIP3P-based protocol has been successfully applied to other Cz systems [25–27]. The RMSD analysis (Fig. 6A) for the alpha-carbon atoms of the protein indicated overall structural stability across all systems, with average values below 1.0 Å: 0.775 ± 0.098 Å (CP3), 0.818 ± 0.091 Å (CP98), 0.813 ± 0.079 Å (LMM1), 0.805 ± 0.084 Å (LMM2), 0.772 ± 0.084 Å (LMM3), and 0.840 ± 0.102 Å (LMM4) (Table 10). These results indicate that the global protein fold remained stable during the simulations, with LMM3 and CP3 showing the lowest average protein Cα RMSD values.

**Table 10:** Structural and dynamic parameters obtained from MD simulations of Cz complexes. RMSD (protein and ligand) and RMSF values are expressed in Ångströms (Å). PC1 and PC2 represent the percentage of each system’s projected conformational variance along the first and second global principal components obtained from the concatenated trajectory analysis. All values represent mean ± standard deviation calculated from three independent replicas of 250 ns each (750 ns total sampling time per system)

System	RMSD (protein)	RMSD (ligand)	RMSF	PC1	PC2
CP3	0.775 ± 0.098	2.592 ± 0.072	0.665 ± 0.071	47.70	36.85
CP98	0.818 ± 0.091	3.208 ± 0.106	0.676 ± 0.045	44.36	30.39
LMM1	0.813 ± 0.079	1.578 ± 0.216	0.645 ± 0.048	59.25	29.58
LMM2	0.805 ± 0.084	2.889 ± 0.098	0.666 ± 0.051	24.90	50.60
LMM3	0.772 ± 0.084	2.123 ± 0.106	0.637 ± 0.040	52.81	26.00
LMM4	0.840 ± 0.102	3.134 ± 0.079	0.674 ± 0.048	58.46	17.14

Because protein alpha-carbon RMSD alone does not demonstrate preservation of the ligand binding mode, ligand heavy-atom RMSD was calculated after protein Cα alignment, using the initial FITTED docking pose as reference. The ligand RMSD values ranged from 1.578 ± 0.216 Å (LMM1) to 3.208 ± 0.106 Å (CP98) (Table 10). LMM1 displayed the lowest ligand RMSD, indicating the smallest rearrangement relative to the initial docking pose. LMM3 and CP3 showed moderate ligand rearrangements, with RMSD values of 2.123 ± 0.106 Å and 2.592 ± 0.072 Å, respectively. LMM2, LMM4, and CP98 exhibited larger deviations, suggesting more pronounced reorientation within the binding pocket. Figure S3 presents the per-replica ligand heavy-atom RMSD time series. As several ligands exhibited rearrangements of approximately 2 to 3 Å relative to the initial docking pose, the MM/GBSA and per-residue decomposition analyses are interpreted as energetic descriptions of MD-relaxed binding modes sampled during the final simulation window, rather than as direct energetic validation of the original docking poses.

The flexibility of the amino acid residues was evaluated using RMSF calculations (Fig. 6B), which indicated similar average values throughout the systems, ranging from 0.637 ± 0.040 Å (LMM3) to 0.676 ± 0.045 Å (CP98) (Table 10). The analysis of the most flexible regions consistently identified movable segments across all systems: Asp57-Gly65 and Glu95-Gly105, which correspond to two loop regions previously recognized as flexible in molecular dynamics studies of Cz [25, 26]. The presence of glycine residues (Gly65 and Gly105) in these regions is noteworthy, as glycine’s lack of a side chain affords greater conformational freedom, thereby enhancing loop flexibility [111]. These regions had RMSF values over 1.0 Å across all systems, indicating significant movements during the simulations while maintaining the overall structural integrity of the Cz systems.

**Fig. 7:**
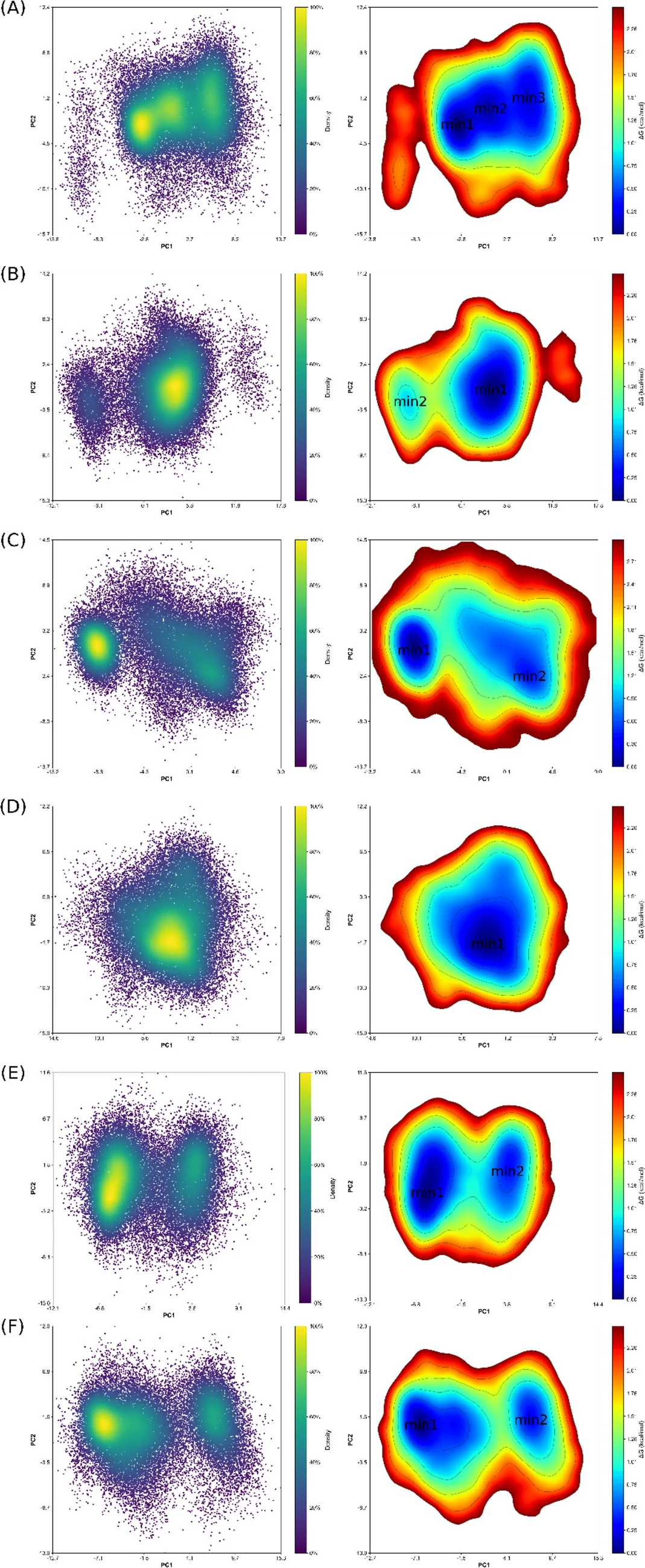
PCA projections (left panels) and FEL (right panels) computed from the first two principal components for the six Cz complexes: **A** CP3, **B** CP98, **C** LMM1, **D** LMM2, **E** LMM3, and **F** LMM4. The FEL color scale ranges from 0 kcal mol^−1^ (blue, global minimum) to 3 kcal mol^−1^ (red). Local minima discussed in the text are highlighted in the FEL panels as min1, min2, and, when present, min3. Data were computed over 750 ns per system, corresponding to three independent replicas of 250 ns each

PCA was employed to investigate the predominant modes of conformational mobility of the protein throughout the MD simulations (Fig. 7, left). The computation was performed over a total duration of 750 ns (3 × 250 ns) for each system’s trajectory. The first principal component (PC1) explained 47.70% (CP3), 44.36% (CP98), 59.25% (LMM1), 24.90% (LMM2), 52.81% (LMM3), and 58.46% (LMM4) of the variance, whereas the second principal component (PC2) accounted for 36.85% (CP3), 30.39% (CP98), 29.58% (LMM1), 50.60% (LMM2), 26.00% (LMM3), and 17.14% (LMM4) of the overall conformational variance (Table 10). For LMM2, the projected variance along global PC2 exceeded that along global PC1 (50.60% compared to 24.90%). This observation does not contradict the variance ordering inherent to principal component analysis, as PC1 and PC2 were derived from the concatenated trajectory dataset and are globally variance-ordered. Instead, these findings suggest that the conformational fluctuations sampled by LMM2 align more closely with the second global collective mode than with the first. The PC1–PC2 projections revealed distinct clustering patterns among the systems, indicating ligand-dependent differences in the conformational sampling of Cz.

The analysis of the FEL using the first two principal components revealed ligand-dependent differences in the conformational sampling of the Cz complexes (Fig. 7, right). CP3 (Fig. 7A) displayed the broadest landscape, with five local minima, three of which were considered thermally accessible and are highlighted as min1–min3. LMM2 (Fig. 7D) sampled a more restricted landscape characterized by a single dominant minimum. CP98 (Fig. 7B), LMM1 (Fig. 7C), LMM3 (Fig. 7E), and LMM4 (Fig. 7F) each displayed two local minima. Because the estimated barriers between these shallow minima were below or close to thermal energy at 310 K, the FELs were interpreted qualitatively rather than as evidence of kinetically well-separated conformational states. Although CP3 was also the most active compound in the phenotypic dataset, the small number of systems analyzed does not allow a robust statistical relationship between the number of FEL minima, barrier heights, and biological activity to be established. Therefore, FEL analysis should be interpreted as a qualitative description of conformational sampling rather than as direct evidence that increased flexibility determines antitrypanosomal potency or Cz inhibition. These results suggest that different pyrazolone derivatives may be associated with distinct conformational substates of Cz, particularly involving flexible regions such as Asp57–Gly65 and Glu95–Gly105.

### MM/GBSA binding free energy estimates and per-residue energy decomposition

The MM/GBSA calculations provided endpoint estimates of the relative binding free energies for the Cz-ligand complexes. In this analysis, LMM2 and LMM4 showed the most favorable computed Δ*G*_bind_ values (− 24.57 and − 24.43 kcal mol^−1^, respectively), followed by LMM1, CP3, CP98, and LMM3 (Table 11). These values should be interpreted as approximate computational estimates rather than direct experimental binding affinities. Decomposition of the energy components indicated that van der Waals interactions were the dominant favorable contribution across the series, whereas polar solvation partially offset electrostatic stabilization, particularly for LMM2 (Δ*E*_vdW_ =  − 35.61 kcal mol^−1^) and LMM4 (Δ*E*_vdW_ =  − 34.45 kcal mol^−1^).

**Table 11:** Binding free energy components from MM/GBSA calculations. *ΔE*_*vdW*_, van der Waals; *ΔE*_*elec*_, electrostatic; *ΔG*_*GB*_, polar solvation; *ΔG*_*np*_, non-polar solvation; *ΔG*_*bind*_, total binding free energy. All values in kcal mol^−1^. The standard deviation values were calculated from three independent replicas of 250 ns each (750 ns total sampling time per system)

System	Δ*E*_vdW_	Δ*E*_elec_	Δ*G*_GB_	Δ*G*_np_	Δ*G*_bind_
CP3	− 30.87 ± 3.47	− 1.40 ± 7.54	14.80 ± 6.75	− 3.62 ± 0.33	− 21.09 ± 3.63
CP98	− 27.91 ± 3.92	− 10.32 ± 8.38	22.03 ± 6.72	− 3.43 ± 0.38	− 19.63 ± 4.30
LMM1	− 29.12 ± 3.95	− 4.03 ± 5.55	14.53 ± 4.73	− 3.65 ± 0.46	− 22.27 ± 4.94
LMM2	− 35.61 ± 2.26	− 14.33 ± 4.20	29.64 ± 3.65	− 4.27 ± 0.21	− 24.57 ± 2.49
LMM3	− 27.32 ± 5.53	− 3.97 ± 7.22	16.19 ± 7.02	− 3.44 ± 0.64	− 18.54 ± 4.84
LMM4	− 34.45 ± 2.47	− 8.56 ± 3.81	22.47 ± 3.14	− 3.89 ± 0.26	− 24.43 ± 2.48

A notable discrepancy was observed between the MM/GBSA ranking and the phenotypic activity trend. Although LMM2 and LMM4 showed more favorable computed Δ*G*_bind_ values than CP3, it remains the most active compound in the original whole-cell dataset, with an experimental pIC_50_ of 6.40. Importantly, CP3 was excluded from the final QSAR regression model as an outlier but was retained as the principal downstream structure-based reference in docking, ADMET, MD, and MM/GBSA analyses because of its high phenotypic potency. In contrast, LMM2 and LMM4 showed only intermediate QSAR-predicted activities. This mismatch indicates that MM/GBSA alone does not reliably reproduce the phenotypic potency ranking of this series [112]. Several factors may contribute to this behavior, including the approximate nature of endpoint free-energy methods, the omission of explicit entropic contributions [72], uncertainties in protonation-dependent electrostatic terms [113], and, importantly, the possibility that Cz is not the sole operative target underlying the observed whole-cell antitrypanosomal activity. Therefore, the subsequent residue decomposition and FEL analyses were used only to generate qualitative structural hypotheses, not to establish a definitive mechanistic explanation for cellular potency.

**Fig. 8:**
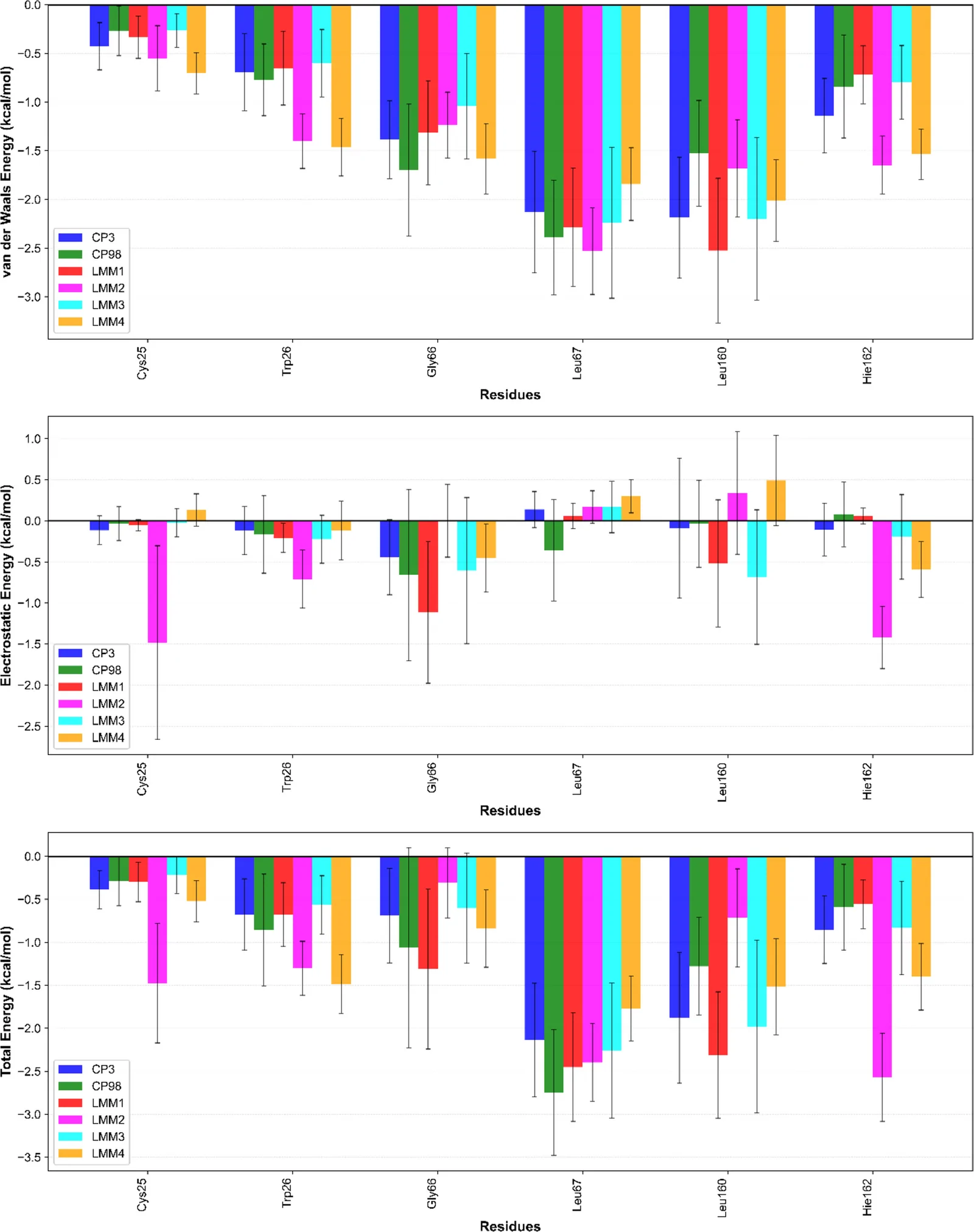
Per-residue energy decomposition (kcal mol^−1^) from MM/GBSA calculations for the six key active-site residues of Cz (Cys25, Trp26, Gly66, Leu67, Leu160, and His162) across all six ligand complexes. Values represent means calculated from the last 20 ns of each of three independent 250 ns replicas

Per-residue MM/GBSA decomposition was performed for selected active-site residues, including Cys25, Trp26, Gly66, Leu67, Leu160, and His162 (Fig. 8). The analysis suggested modest differences in residue-level energetic contributions among the complexes. For example, Gly66, located near the S3 subsite, contributed more favorably to CP3 than to LMM2. Because the magnitude of this difference is small and within the expected uncertainty of endpoint decomposition methods, it should not be interpreted as a quantitative determinant of potency. Nevertheless, the recurrent involvement of Gly66 is structurally relevant because this region has been associated with Cz recognition and selectivity over related human cathepsins [40, 114].

The S2 subsite, represented here by Leu67 and Leu160, also showed modestly different energetic contributions across the series. CP3 exhibited a somewhat more favorable combined contribution from these residues than LMM2 and LMM4. This observation is consistent with the known relevance of hydrophobic complementarity within the Cz S2 pocket [101], but the magnitude of the differences is insufficient to establish this term as a quantitative predictor of phenotypic activity. Thus, S2 interactions should be viewed as a plausible structural feature to consider in future optimization, rather than as a definitive explanation for the activity ranking.

The favorable binding energy of LMM2 was primarily affected by exceptionally strong electrostatic interactions with the residues of the catalytic dyad. LMM2 demonstrated electrostatic contributions of − 1.48 kcal mol^−1^ with Cys25 and a cumulative energy of − 2.57 kcal mol^−1^ with His162. However, these beneficial electrostatic interactions were counterbalanced by a considerable desolvation penalty (Δ*G*_GB_ =  + 29.64 kcal mol^−1^), approximately double that observed for CP3 (+ 14.80 kcal mol^−1^). For LMM2, the favorable computed Δ*G*_bind_ appears to arise partly from strong electrostatic contributions involving the catalytic dyad region, which are substantially offset by a large polar desolvation penalty. In parallel, FEL analysis showed a more restricted conformational landscape for this complex. These features may contribute to the discrepancy between the favorable MM/GBSA estimate and the moderate predicted phenotypic activity of LMM2. However, this interpretation remains qualitative, particularly because the entropic term was not included in MM/GBSA and because the biological pIC_50_ values reflect whole-cell antitrypanosomal activity rather than direct Cz inhibition. This observation is consistent with a broader pattern emerging from experimental studies on pyrazole-class compounds against *T. cruzi*. De Oliveira et al. [24] demonstrated that structural optimization of a pyrazole-imidazoline scaffold to enhance antiparasitic potency simultaneously abolished its ability to inhibit *T. cruzi* cysteine proteinase activity, with the most active derivative (IC_50_ = 3.3 µM against intracellular amastigotes) showing only 3.76% Cz inhibition at 300 µM. Taken together, our computational results and the biochemical findings reported by de Oliveira et al. [24] suggest that whole-cell antiparasitic potency within pyrazole-related chemotypes is not necessarily a direct function of global Cz binding affinity. In the absence of biochemical Cz inhibition data for the present pyrazolone series, the current structure-based results should therefore be interpreted as hypotheses about possible Cz recognition rather than as proof of the molecular origin of cellular activity. From this perspective, interactions with S2/S3 residues, the thermodynamic balance between favorable interactions and desolvation costs, and conformational adaptability may serve as useful criteria for future experimental evaluation, but their role in Cz inhibition remains to be confirmed.

In contrast, CP3 showed a more balanced energy profile, characterized by considerable contributions from both van der Waals and electrostatic interactions, resulting in a lower polar desolvation penalty than LMM2. Moreover, CP3 maintains beneficial interactions with residues located in the S2/S3 subsites, which may be relevant for Cz recognition. The FEL analysis indicated broader conformational sampling for CP3, with five local minima, three of which were considered thermally accessible. However, because CP3 was excluded from the final QSAR model and because the phenotypic pIC_50_ values do not directly measure Cz inhibition, these observations should be interpreted as qualitative structural hypotheses rather than as determinants of inhibitory potency.

Overall, the MM/GBSA, per-residue decomposition, and FEL analyses indicate that global endpoint binding energies should not be used as the sole criterion to prioritize this pyrazolone series. The lack of agreement between Δ*G*_bind_ and phenotypic pIC_50_ values suggests that either MM/GBSA has limited ranking power for these compounds or that Cz binding alone does not explain the whole-cell antitrypanosomal response. Within these limitations, the structural analyses point to S2/S3 subsite contacts, particularly involving Leu67, Leu160, and Gly66, as plausible features for future optimization and biochemical testing. Therefore, the proposed compounds should be regarded as antitrypanosomal pyrazolone derivatives with computationally predicted Cz-binding modes, rather than experimentally validated Cz inhibitors. Furthermore, because several ligands underwent rearrangements relative to their initial docking poses, the residue decomposition should be interpreted as describing MD-relaxed binding modes sampled during the final simulation window rather than the original docking hypotheses.

A limitation of this study is that we did not include entropy in our MM/GBSA calculations. The omission of configurational entropy is particularly relevant because conformational flexibility, characterized here using PCA and FEL analyses, may influence ligand binding. Consequently, the present calculations cannot quantify the entropic contribution to binding. More direct tests, such as biochemical Cz inhibition assays, enhanced-sampling simulations, and rigorous free-energy calculations, are needed to determine whether the pyrazolone derivatives inhibit Cz and whether the observed conformational features affect binding affinity or selectivity.

## Conclusions

This study employed an integrated computational framework combining 2D-QSAR modeling, molecular docking, MD simulations, binding free energy calculations, and in silico ADMET predictions to evaluate pyrazolone derivatives with reported antitrypanosomal activity and their computationally predicted interaction with Cz. The final 2D-QSAR model demonstrated satisfactory statistical performance and external predictive ability within its applicability domain, identifying molecular descriptors associated with whole-cell antitrypanosomal activity. These insights directly informed the design of four new derivatives (LMM1–LMM4), which showed intermediate predicted antitrypanosomal activity within the chemical space of the original pyrazolone series. Therefore, these candidates were proposed based on structural novelty, acceptable predicted activity, and favorable preliminary drug-likeness/ADMET profiles, rather than superior predicted potency relative to the most active compounds in the original dataset. Molecular docking suggested favorable predicted binding poses within the Cz active site, with contacts involving residues in the S2 and S3 subsites. Extended MD simulations indicated overall structural stability of the Cz complexes, while PCA and FEL analyses revealed ligand-dependent conformational sampling. However, the limited number of systems analyzed does not allow a definitive correlation between conformational flexibility and phenotypic potency to be established. MM/GBSA calculations indicated that van der Waals interactions are major contributors to the computed binding energies, but the Δ*G*_bind_ ranking did not reproduce the whole-cell activity trend. This discrepancy highlights the limitations of endpoint-binding free-energy methods for this series and reinforces the notion that phenotypic antitrypanosomal potency cannot be directly assigned to Cz inhibition without biochemical validation. Per-residue decomposition suggested that contacts with Gly66, Leu67, and Leu160 may be relevant for Cz recognition, but these contributions should be interpreted qualitatively. Because several ligands underwent rearrangements relative to their initial docking poses, MM/GBSA and per-residue decomposition analyses should be interpreted as energetic descriptions of MD-relaxed binding modes rather than direct validation of the original docking hypotheses. In silico ADMET profiling further supported the drug-likeness of the proposed derivatives, indicating favorable predicted pharmacokinetic properties and low predicted toxicity, strengthening their candidacy for further experimental evaluation. Finally, the results suggest that hydrophobic contacts within the S2/S3 subsites, balanced energetic contributions, and ligand-dependent conformational adaptability may influence the predicted interaction of pyrazolone derivatives with Cz. Nevertheless, because the QSAR model was based on whole-cell antitrypanosomal activity and no biochemical Cz inhibition data are available for the proposed compounds, these findings should be considered structural hypotheses requiring experimental validation. This study therefore provides a computational framework for prioritizing antitrypanosomal pyrazolone derivatives and guiding future Cz-directed biochemical assays.

## Supplementary information

Below is the link to the electronic supplementary material.

ESM 1 (ZIP 34.0 KB)

ESM 2 (PDF 731 KB)

## Data Availability

All data supporting the findings of this study are provided in the manuscript, the Supporting Information, and a public GitHub repository: https://github.com/jrasilva-ufpa/cruzain-pyrazolone-cadd. Additional information can be obtained from the corresponding author upon reasonable request.
