# Supplementary material for: Molecular modeling design of antitrypanosomal pyrazolone derivatives targeting Chagas disease: QSAR, docking, molecular dynamics and free energy calculations
Source: J Mol Model. 2026 Sep 9;32(10):357. doi: 10.1007/s00894-026-06923-0 (PMC13558299; doi:10.1007/s00894-026-06923-0)

Molecular Modeling Design of Antitrypanosomal Pyrazolone Derivatives Targeting Chagas Disease: QSAR, Docking, Molecular Dynamics and Free Energy Calculations

Karen Karla F. Sousa, Ana Beatriz S. Barbosa, Fábio José B. Cardoso, José Rogério A. Silva and Fábio A. Molfetta**

Supporting Information

Table S1. SMILES codes of compounds synthesized experimentally by Sijm et al.

ID	SMILES
3	c1ccncc1c1cc(ccc1OC)C1=NN(C(=O)C1(C)C)C(C)C
57	c1ccncc1c1cc(cc(c1)OC)C1=NN(C(=O)C1(C)C)C(C)C
58	c1ccncc1c1cc(cc(c1OC)OC)C1=NN(C(=O)C1(C)C)C(C)C
59	c1ccncc1c1cc(cc(c1)CCOC)C1=NN(C(=O)C1(C)C)C(C)C
60	c1ccncc1c1cc(cc(c1OC)Br)C1=NN(C(=O)C1(C)C)C(C)C
61	c1ccncc1c1cc(ccc1)C1=NN(C(=O)C1(C)C)C(C)C
62	c1ccncc1c1cc(ccc1F)C1=NN(C(=O)C1(C)C)C(C)C
63	c1ccncc1c1cc(ccc1Cl)C1=NN(C(=O)C1(C)C)C(C)C
64	c1ccncc1c1cc(ccc1C)C1=NN(C(=O)C1(C)C)C(C)C
65	c1ccncc1c1c(c(ccc1OC)C1=NN(C(=O)C1(C)C)C(C)C)F
66	c1ccncc1c1cc(c(cc1OC)F)C1=NN(C(=O)C1(C)C)C(C)C
67	c1ccncc1c1cc(cc(c1OC)C)C1=NN(C(=O)C1(C)C)C(C)C
68	c1c(OC)c(cc(C2=NN(C(C)C)C(=O)C2(C)C)c1C)c1cccnc1
74	c1ccncc1c1cc(ccc1C#N)C1=NN(C(=O)C1(C)C)C(C)C
89	c1ccncc1c1cc(ccc1OC)C1=NN(C(=O)C21CCCC2)C(C)C
90	c1ccncc1c1cc(ccc1OC)C1=NN(C(=O)C21CC=CC2)C(C)C
91	c1ccncc1c1cc(ccc1OC)C1=NN(C(=O)C21CCN(CC2)C)C(C)C
92	c1ccncc1c1cc(ccc1OC)C1=NN(C(=O)C21CCOCC2)C(C)C
95	c1ccncc1c1cc(ccc1OC)c1nn2c(c1)OCC2
98	c1ccncc1c1cc(ccc1OC)Cc1nnc(s1)N
99	c1ccncc1c1cc(ccc1OC)C1=NN(C(=S)C1(C)C)C(C)C
104	c1ccncc1c1cc(ccc1OC)C1=NN(C(=O)CC1)C(C)C
109	c1ccncc1c1cc(ccc1OC)C1=Nn2c(C1(C)C)nnc2C1CC1
120	c1ccncc1c1cc(ccc1OC)C1NC([CH]O1)C(C)(C)C
121	c1ccncc1c1cc(ccc1OC)C1NC([CH]O1)c1ccccc1
122	c1ccncc1c1cc(ccc1OC)C1NC([CH]O1)c1cccc(c1)F
123	c1ccncc1c1cc(ccc1OC)C1NC([CH]O1)c1ccc(cc1)F
124	c1ccncc1c1cc(ccc1OC)C1NC(C(O1)C)c1ccccc1
132	c1ccncc1c1cc(ccc1OC)C1OC(NN1)C(C)C
133	c1ccncc1c1cc(ccc1OC)C1OC(NN1)C1CCCC1
134	c1ccncc1c1cc(ccc1OC)C1OC(NN1)CC1CCOCC1
135	c1ccncc1c1cc(ccc1OC)C1OC(NN1)c1ccccc1
136	c1ccncc1c1cc(ccc1OC)C1OC(NN1)c1cccc(c1)F
141	c1ccncc1c1cc(ccc1OC)C1NC([CH]S1)C1CC1
142	c1ccncc1c1cc(ccc1OC)C1NC([CH]S1)c1ccccc1

Table S2 Validation Parameters for the 33- and 35-compounds 2-D QSAR models

Statistical Parameters	VALUES	
	**The final model with 33 compounds**	**The initial model 35 with compounds**
3 LVs	73.09%	72.37%
k	5	5
n	23	24
R^2	0.892	0.833
Q^2_{LOO}	0.805	0.741
R^2 - Q^2_{LOO}	0.087	0.092
$PRESS_{cal}$	0.89	1.608
$PRESS_{val}$	1.612	2.501
SS_y	8.278	9.638
$PRESS_{cal}/SS_y$	0.108	0.167
$PRESS_{val}/SS_y$	0.195	0.259
$RMSE_{val}$	0.265	0.323
$RMSE_{cal}$	0.197	0.259
$R^2_{adjusted}$	0.875	0.817
R^2 - $R^2_{adjusted}$	0.017	0.016
$F_{(n,\ n\text{-}k\text{-}1)\text{calc}}$	52.31	33.25
$F_{(n,\ n\text{-}k\text{-}1)}$ reference value	3.13	3.10
$F_{(n,\ n\text{-}k\text{-}1)\text{calc}}$/ $F_{(n,\ n\text{-}k\text{-}1)}$reference value	16.71	10.33

Fig S1 RMSD (top) and Radius of Gyration (bottom) profiles for apo-Cz systems using ff14sb (black) and ff19sb (red).

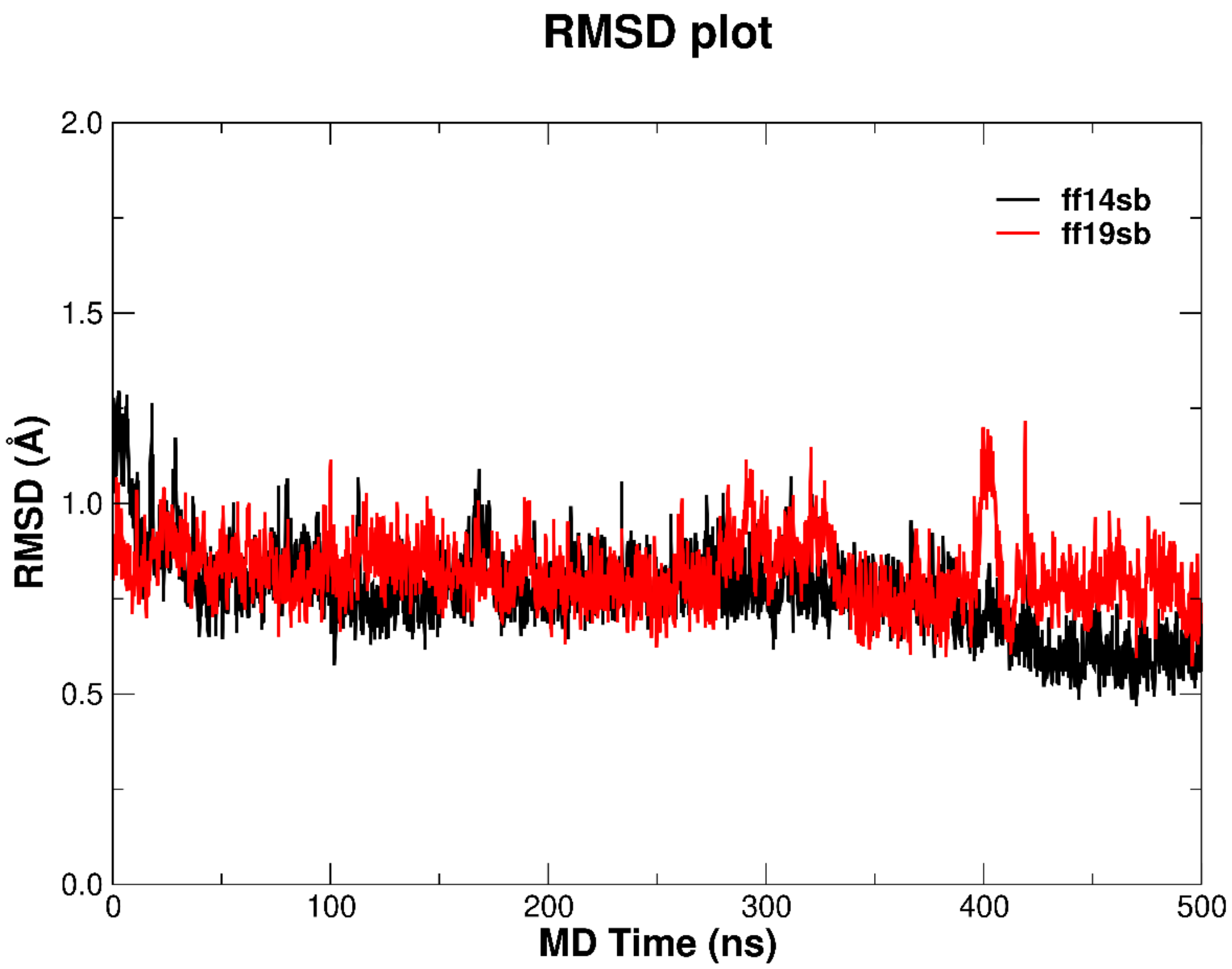

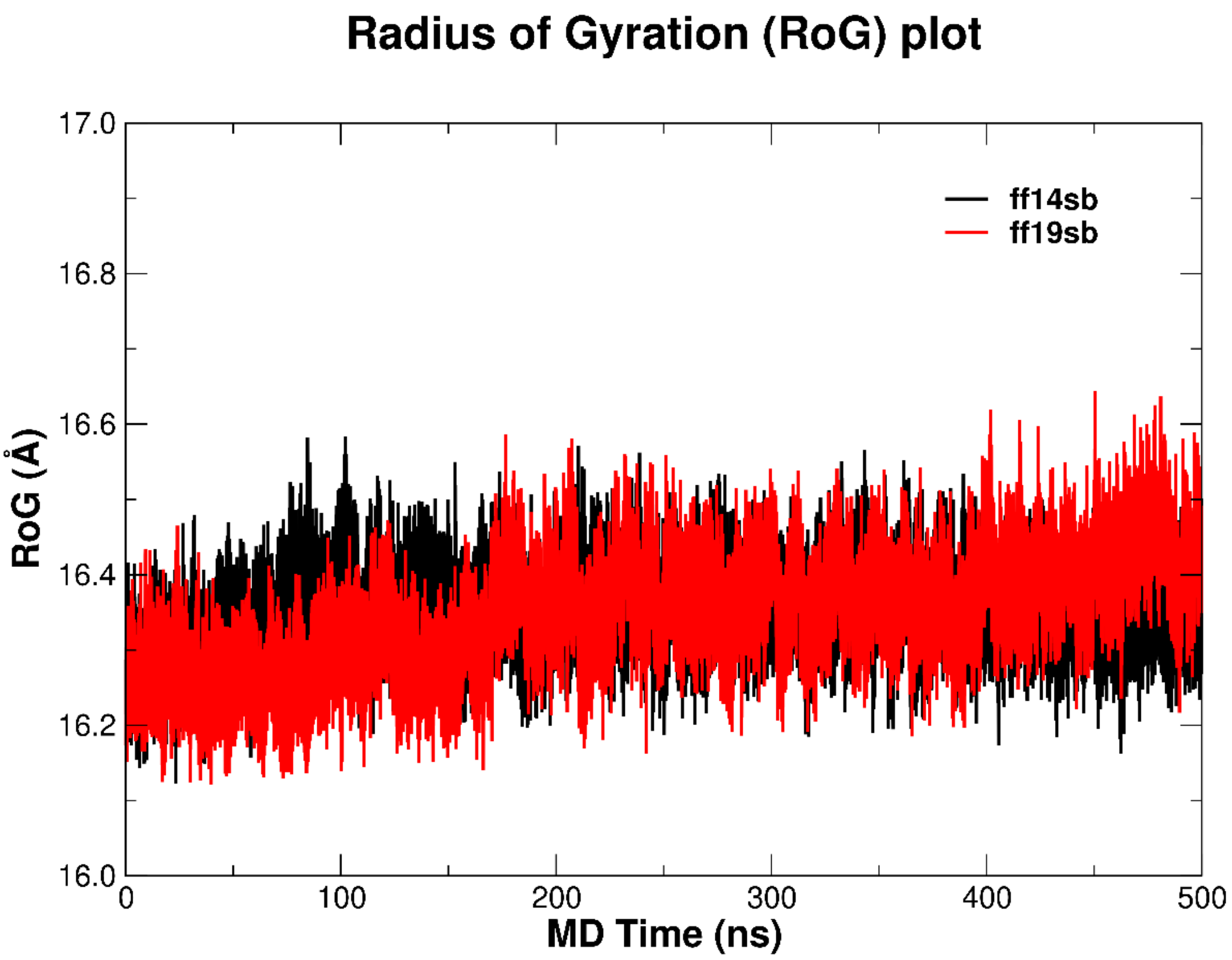

Fig S2 3D conformations of LMM1 (green), LMM2 (cyan), LMM3 (purple) and LMM4 (yellow) compounds from docking results.

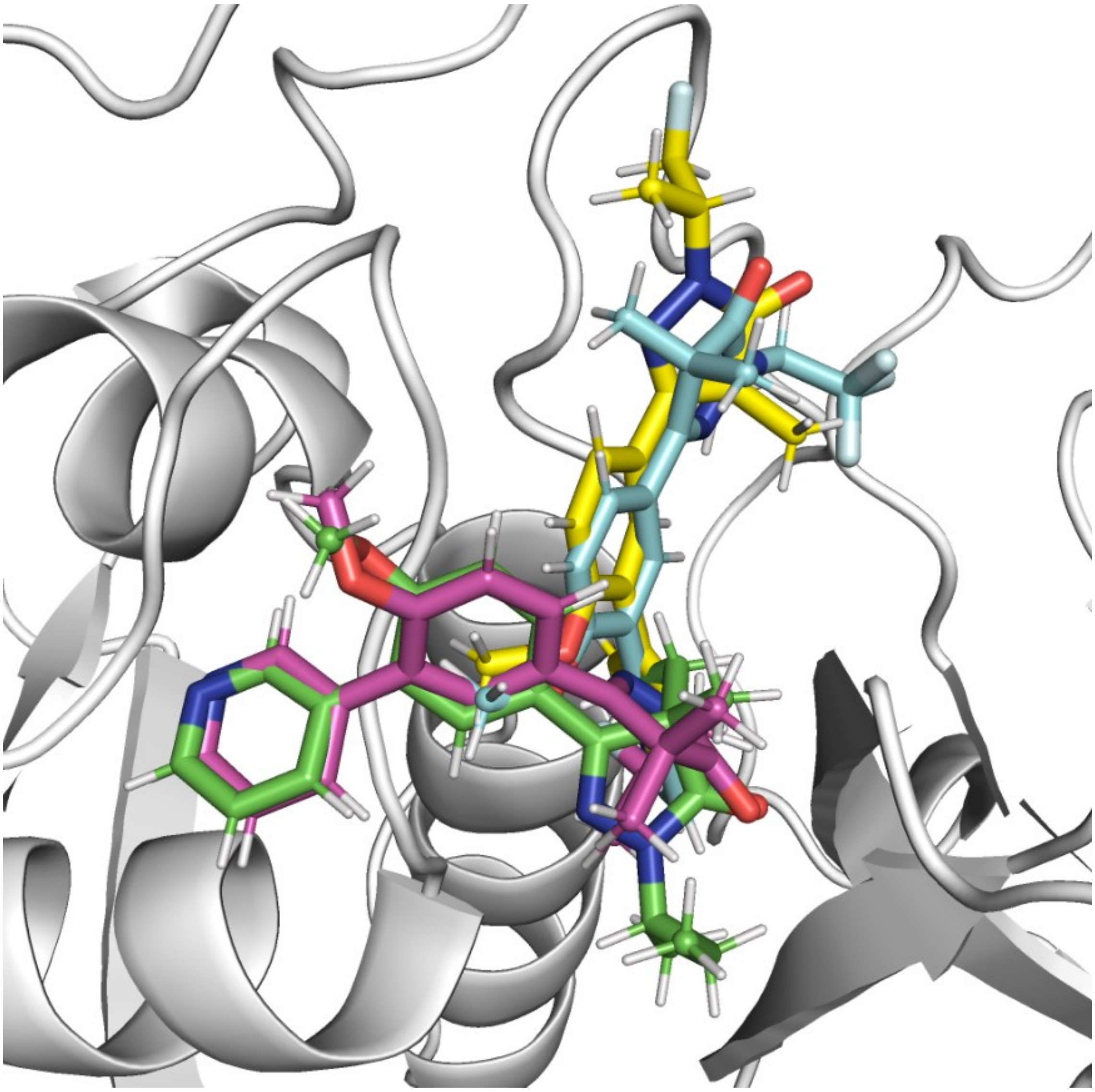

Fig S3 Ligand heavy-atom root mean square deviation (RMSD) for the six Cz–ligand complexes. RMSD values were calculated after least-squares fitting of the protein Cα atoms to the initial complex structure, using the initial FITTED docking pose as reference. Data were computed from three independent replicas of 250 ns for each system.

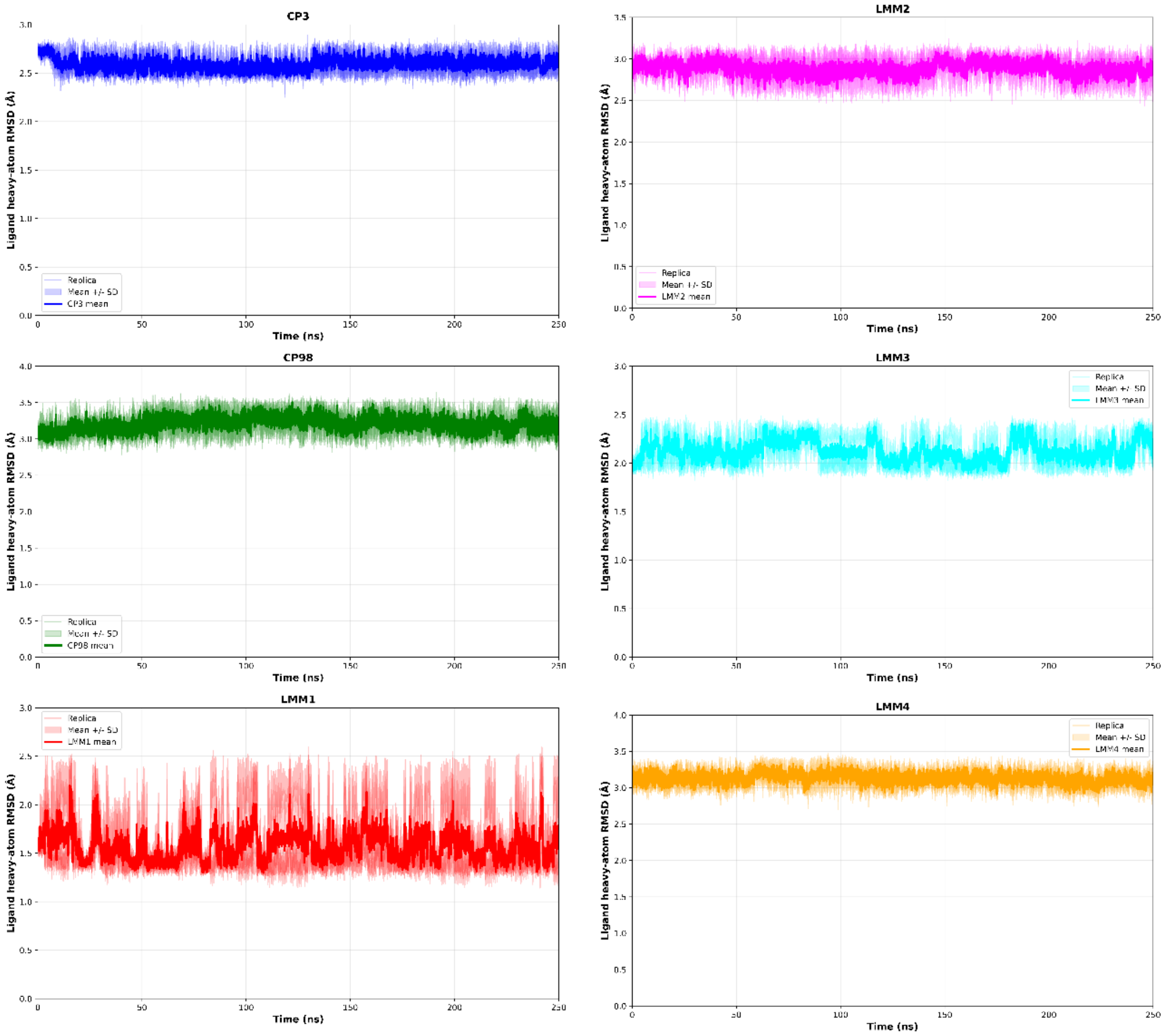

Supplement: Supplementary file 1 [file 894_2026_6923_MOESM2_ESM.pdf]
